# Integrated multi-omic analyses reveal novel gene–metabolite relationships in human steatohepatitic hepatocellular carcinoma

**DOI:** 10.1016/j.jlr.2026.101081

**Published:** 2026-06-17

**Authors:** Garrett B. Anspach, Robert M. Flight, Sehyung Park, Daheng He, Chi Wang, Hunter N.B. Moseley, Robert N. Helsley

**Affiliations:** 1 Barnstable Brown Diabetes Center, University of Kentucky College of Medicine, Lexington, Kentucky, USA; 2 Markey Cancer Center, University of Kentucky College of Medicine, Lexington, Kentucky, USA; 3 Saha Cardiovascular Research Center, University of Kentucky, Lexington, Kentucky, USA; 4 Division of Endocrinology, Diabetes, and Metabolism, Department of Internal Medicine, University of Kentucky College of Medicine, Lexington, Kentucky, USA; 5 Department of Molecular and Cellular Biochemistry, University of Kentucky College of Medicine, Lexington, Kentucky, USA; 6 Biostatistics and Bioinformatics Shared Resource Facility, Markey Cancer Center, University of Kentucky, Lexington, Kentucky, USA

**Keywords:** Hepatocellular carcinoma, Metabolic dysfunction-associated steatotic liver disease, Fatty acids, Liver cancer, Lipidomics

## Abstract

Metabolic dysfunction-associated steatotic liver disease (MASLD) is the fastest-growing etiology of hepatocellular carcinoma (HCC). Understanding the metabolic heterogeneity of MASLD-driven tumors is crucial to inform strategies for future treatment options. Paired tumor (n = 8) and adjacent non-tumor tissue (n = 8) were collected from patients with steatohepatitic HCC at the University of Kentucky Markey Cancer Center. Hematoxylin and eosin (H&E) staining was used for pathological determination of tumor and adjacent nontumor tissue by a board-certified pathologist. Lipidomic, metabolomic, and transcriptomic analyses were performed, and data were integrated across platforms to identify novel relationships across tumor and adjacent nontumor tissue. Paired transcriptomic analyses were validated in 424 human samples from The Cancer Genome Atlas-Liver Hepatocellular Carcinoma (TCGA-LIHC). Histological analysis by H&E showed significant lipid vacuole accumulation and inflammatory foci in HCC tumors relative to nontumor tissue. Across omics platforms, we identified 1,679 genes, 1,696 metabolites, and 292 lipids that were significantly (*p*adj < 0.01) increased or decreased across all paired (tumor vs. nontumor) patient samples. We identified significant reductions in ceramides and linoleic acid-enriched lipids, and increases in fatty acyl chain saturation in tumor tissue. Metabolites involved in purine and fatty acid catabolism were commonly decreased in tumors relative to nontumor tissue across paired samples. We also identified a total of 303 highly significant and novel transcript-metabolite associations (117 gene-metabolite; 186 gene-lipid) across tumor and nontumor tissue. Taken together, this integrative analysis reveals novel relationships between steady-state gene transcripts and specific metabolites in steatohepatitic tumors, thereby identifying new pharmacological targets that may be exploited for therapeutic benefit.

Hepatocellular carcinoma (HCC) is the most common form of liver cancer worldwide, accounting for ∼90% of all liver-related cancers (1). Current projections estimate that more than 1 million individuals are affected by HCC annually (1). The prognosis of HCC associates closely with tumor stage, with early stage diagnoses associating with greater 5-years survival rates at ∼70%, while more advanced HCCs associate with rates as low as ≤20% (2, 3). Survival rates also depend on tumor heterogeneity, etiology, treatment, and patient comorbidities (4). Viral-induced hepatitis remains the foremost risk factor for HCC; however, the prevalence of metabolic dysfunction-associated steatotic liver disease (MASLD) is rapidly escalating and emerging as a prominent etiology of HCC. In the United States from 2004 to 2009, MASLD-driven HCC showed a 9% annual increase with ∼14% of all HCC cases being attributed to MASLD (5). Across Asian countries, it is estimated that HCC will increase by 44%–85% from 2016 to 2030 in large part due to non-viral factors, such as MASLD and its comorbidities (obesity, diabetes, and hyperlipidemia) (6). This estimated global shift in HCC etiology from viral to non-viral sources warrants identification of novel pathways in MASLD-HCC pathogenesis that may be exploited for therapeutic benefit.

The World Health Organization approximates that 35% of all HCC cases can be histologically classified into one of eight subtypes. The most common subtype is Steatohepatitic Hepatocellular Carcinoma (SH-HCC), accounting for up to 20% of all cases (7, 8). Histologically, SH-HCC often reflects that of MASLD and metabolic dysfunction-associated steatohepatitis (MASH) in the nonneoplastic liver, including the presence of steatosis, cell ballooning, inflammation, fibrosis, and Mallory-Denk bodies (7, 8). The SH-HCC variant was diagnosed in ∼36% of patients with confirmed MASH or MASLD with increased alcohol intake (MetALD), as compared to ∼1% of individuals with other liver disease etiologies (9). Consistent with a MASLD-driven etiology, approximately 50% of SH-HCC patients had at least 3 metabolic syndrome components, as compared to 22.5% with other etiologies (9). Despite the increased associations with metabolic syndrome, 5-year survival rates of patients with SH-HCC are comparable to that of patients with non-SH-HCC (10). Other unique histological features of SH-HCC, as compared to conventional HCC, include the number of activated stellate cells and subsequent fibrosis within the tumor core, as well as loss of cytokeratin 8/18 expression within ballooned hepatocytes of SH-HCC (9). While SH-HCC represents an increasingly prevalent subtype of HCC, identification of the metabolic heterogeneity of SH-HCC tumors is crucial to inform strategies for future treatment options.

In this article, we collected eight paired tumor and adjacent nontumor tissues from patients with biopsy-confirmed HCC who met at least one of five cardiometabolic risk factors or who met the cutoff for MetALD (11), and exhibited a tumor microenvironment consistent with that of SH-HCC by histological analysis. We then employed omics-based techniques to identify novel relationships between RNA transcripts, lipids, and metabolites that were commonly increased or decreased across all patient paired tumor and adjacent nontumor tissue. We report significant alterations in ceramides, lipid saturation, and purine and fatty acid metabolism, with a total of 303 highly significant transcript-metabolite associations identified across paired tissue. This methodological approach offers in-depth pairwise analyses of integrated data across omics platforms, thereby aiding in the identification of novel pathways controlling cancer progression.

## Materials and methods

### Human tissue collection

Patients undergoing surgical resection of primary HCC at the University of Kentucky. All patients were consented for tissue donation under the approved University of Kentucky institutional review board protocols #44026 and #70872. All human studies abided by the ethical principles set forth by the Declaration of Helsinki. Surgical resection specimens were collected as part of routine patient care and sent by the surgeon to UK surgical pathology for dissection and assessment. Tumor and non-tumor adjacent tissue were then provided to the Biospecimen Procurement and Translational Pathology (BPTP) Shared Resource Facility of Markey Cancer Center staff. Samples were aliquoted, flash frozen, and then stored in vapor-phase liquid nitrogen freezers in the BPTP. Aliquots were transferred on dry ice to the BPTP histology laboratory, embedded in OCT, and sectioned for hematoxylin and eosin (H&E) staining. The H&E slides were reviewed by an independent pathologist to identify tumor versus adjacent non-tumor tissue. Deidentified tissue samples were then provided to the researchers for bulk RNA-sequencing, lipidomic, and metabolomic analyses. An experimental overview is provided in Supplemental Fig. S1.

### Bulk RNA-Sequencing

RNA was isolated from paired tumor and adjacent nontumor tissue via the RNeasy Mini kit (Qiagen). The quantity and quality of the samples were determined using the Cytation 5 (BioTek) plate reader and Agilent 4150 Tape Station System, respectively. RNA that did not meet an RNA integrity cutoff of 4.0 were not included in the analysis (Supplemental Table S1). mRNA-seq libraries and paired-end sequencing were all performed by Novogene, as previously described (12, 13).

### Immunoblotting

Immunoblotting was performed as previously described (12, 13, 14, 15). In short, tumor and adjacent nontumor tissue (∼10–20 mg) were solubilized in 1 ml of 1X ice-cold radioimmunoprecipitation assay buffer (Cell Signaling #9806) supplemented with 1X protease (Selleck Chemicals #B14001) and 1X phosphatase inhibitor cocktail (Selleck Chemicals #B15001-A, B15001-B). The supernatant was collected after 2-sequential centrifugation steps (16,000 × *g* for 10 min at 4°C), and protein was quantified using the Pierce Bicinchoninic Acid Protein assay (ThermoFisher #23225). For SDS-PAGE, 10 μg of protein was loaded and separated on a 4%–20% gradient criterion tris-glycine gel (Bio-Rad #5671095). Proteins were blocked with 5% milk (Bio-Rad #1706404) and probed with the following 1° antibodies overnight at 4°C: ACSL4 (1:10,000; Proteintech #81196-1-RR), SQLE (1:1,000; Proteintech #12544-1-AP), and Vinculin (1:1,000; Novus Biologicals #NB600-1293). All 1° antibodies were diluted in 3% BSA. The following secondary antibodies were diluted in 5% milk at 1:10,000: Peroxidase AffiniPure Goat Anti-Rabbit IgG (Jackson ImmunoResearch #111-035-144) and Peroxidase AffiniPure Goat Anti-Mouse IgG (Jackson ImmunoResearch #115-035-003). Images were taken on a ChemiDoc MP Imaging System (Bio-Rad).

### The Cancer Genome Atlas liver hepatocellular carcinoma (TCGA-LIHC)

RNA-sequencing gene expression data of 374 tumor samples (from 371 patients) and 50 matched normal samples from TCGA-LIHC (liver hepatocellular carcinoma) were downloaded from the Genomic Data Commons (https://gdc.cancer.gov). Associated clinical data were obtained from cBioPortal (https://www.cbioportal.org). A fitted linear mixed model was used to compare gene expression between tumor and normal tissues. To assess whether tumor–normal differences in gene expression varied by sex, we extended the model to include sex, tissue type, and their interaction term. Sex-dependent effects were evaluated by testing the interaction. Resulting *P*-values were corrected for multiple comparisons using the Benjamini–Hochberg procedure to control the false discovery rate (FDR).

### University of California (UC) West Coast Metabolomics

Tissue sample extractions, lipidomic and metabolomic analyses were performed by the UC Davis West Coast Metabolomics Center. Extraction for lipids and polar hydrophilic small molecules were performed using a previously established methyl-tert-butyl ether (MTBE) protocol (16). In short, 4 mg of liver tissue was weighed and 225 μl of ice-cold methanol containing an internal standard mixture was added with 750 μl of MTBE. Samples were then vortexed for 10s and mixed by shaking for 5 min at 4°C. After, 188 μl LC-MS grade H2O was added, samples were then vortexed for 20 s and centrifuged for 2 min at 14,000 × rpm for phase separation. The organic (upper; 350 μl) phase and the aqueous (bottom; 110 μl) phase were separated into individual tubes and dried down for reverse-phase liquid chromatography-high resolution tandem mass spectrometry (LC-HRMS/MS) for lipidomics, hydrophilic interaction chromatography (HILIC) LC-HRMS/MS for biogenic amines, and gas chromatography-time-of-flight mass spectrometry (GC-TOFMS) for the primary metabolism platform.

#### Lipidomics

After dry down, samples were reconstituted in 110 μl MeOH:Tol (9:1) + 12-cyclohexylamino-carbonyl-amino-dodecanoic acid (CUDA; 50 ng/ml) + Avanti SPLASHone lipidomics internal standard mixture. Samples were vortexed for 10s, sonicated for 5 min, and centrifuged for 2 min at 16,000 × *g*. Fifty μL was transferred to two separate glass amber vials with microinserts for positive and negative ionization modes.

##### Positive ionization mode

Mobile phase consists of A: 60:40 ACN:H_2_O+10 mM ammonium formate+0.1% formic acid and B: 90:10 IPA:ACN+10 mM ammonium formate+0.1% formic acid. The column used was an Acquity Premier BEH C18 1.7 μm, 2.1 × 50 mm with an injection volume of 1 μl. Samples were injected using Agilent 6530 QTOF collecting data from 120 m/z to 1200 m/z at an acquisition rate of 2 spectra/sec.

##### Negative ionization mode

Mobile phase consists of A: 60:40 ACN:H_2_O+10 mM ammonium acetate and B: 90:10 IPA:ACN+10 mM ammonium acetate. The column used was an Acquity Premier BEH C18 1.7 μm, 2.1 × 50 mm with an injection volume of 5 μl. Samples were injected using Agilent 6550 QTOF collecting data from 60 m/z to 1200 m/z at an acquisition rate of 2 spectra/sec.

##### Data Processing

The general workflow for data processing was completed using MS-DIAL (17), followed by data cleanup using MS-FLO (18). In brief, raw files were first converted via Abf Converter and processed using MS-DIAL. Once the results were exported from MS-DIAL, a blank reduction was done based on the max peak height relative to the blank average height. Using MS-FLO, potential duplicates and isotopes were checked and deleted if identified. Next, MS/MS spectra were assessed before combining adducts. Peaks were annotated in manual comparison of MS/MS spectra and accurate masses of the precursor ion to spectra given in the Fiehn laboratory’s LipidBlast spectral library (19). Additional peaks were found by manual curation of sample chromatograms on a scan-by-scan basis. MassHunter Quant software was then used to verify peak candidates based on peak shape, peak height reproducibility, and retention time reproducibility in replicate samples. Valid and reproducible peaks were confirmed with MS/MS spectra with the aim of increasing overall peak annotations in both positive and negative modes. These manually curated compounds were incorporated into a.txt file that had a list of accurate masses and retention times for the lipidomics platform. All data were normalized to the sum of internal standards and are reported as peak heights for quantification ion at a specific retention time. Peak heights are used for two reasons: (1) they are more precise for low-abundant metabolites than peak areas, due to the larger influence of baseline determinations on areas compared to peak heights, and (2) co-eluting ions or peaks are harder to deconvolute on peak areas than on peak heights.

#### Biogenic amines and small-molecule metabolites

After dry down, samples were reconstituted in 100 μl of 80:20 ACN:H_2_O + internal standard mixture. The samples were then vortexed for 10s, sonicated for 5 min, and centrifuged for 2 min at 16,000 × *g*. 45 μl was transferred to two separate glass amber vials with micro inserts for positive and negative ionization modes. All data were normalized to the sum of internal standards.

##### Positive ionization mode

Mobile phase consists of A: 100% H_2_O + 10 mM ammonium formate + 0.125% formic acid and B: 95:5 ACN:H_2_O + 10 mM ammonium formate + 0.125% formic acid. The column used was an Acquity Premier UPLC BEH Amide 1.7 μm, 2.1 × 50 mm with an injection volume of 1 μl. Samples were injected into an Agilent 1290 UHPLC/Sciex TripleTOF 6600 mass spectrometer.

##### Negative ionization mode

Mobile phase consists of A: 100% H_2_O + 10 mM ammonium formate + 0.125% formic acid and B: 95:5 ACN:H_2_O + 10 mM ammonium formate + 0.125% formic acid. The column used was an Acquity Premier UPLC BEH Amide 1.7 μm, 2.1 × 50 mm with an injection volume of 1 μl. Samples were injected into an Agilent 1290 UHPLC/Sciex TripleTOF 6600 mass spectrometer.

##### Data Processing

Chromatograms first underwent a quality control check in which internal standards were examined for consistency of peak height and retention time. Raw data files were then processed using an updated version of MS-DIAL software (17), which identified and aligned peaks and then annotated those peaks using both an in-house mzRT library and MS/MS spectral matching with NIST/MoNA libraries. All MS/MS annotations were then manually curated to ensure that only high-quality compound identifications were included in the final analysis.

#### Primary metabolites

Samples are shaken at 30°C for 1.5 h. Then, 91 μl of N-trimethylsilyl-N-methyl trifluoroacetamide (MSTFA) + fatty acid methyl esters (FAMEs) internal standards are added to each sample and are shaken at 37°C for 0.5 h to finish derivatization. Samples are then vialed, capped, and injected onto the 7890A GC coupled with a LECO TOF. Derivatized sample volume is 0.5 uL using a split-less method onto a RESTEK RTX-5SIL MS column with an Integra-Guard at 275C with a helium flow rate of 1 ml/min. The GC oven was set to hold at 50°C for 1 min, then ramp to 20°C/min to 330°C and then hold for 5 min. The transfer line was set to 280°C while the EI ion source was set to 250°C. The mass spectrometer parameters have been published elsewhere (20) and collect data from 85 m/z to 500 m/z at an acquisition rate of 17 spectra/sec.

##### Data Processing

ChromaTOF 4.72 was used for data preprocessing without smoothing, 3 s peak width, baseline subtraction just above noise level, and automatic mass spectral deconvolution and peak detection at signal/noise levels of 5:1 throughout the chromatogram. Apex masses were reported for use in the BinBase algorithm. Result ∗.txt files were then exported to a data server with absolute spectra intensities and further processed by a filtering algorithm implemented in the metabolomics BinBase database. The BinBase algorithm (rtx5) used the settings: validity of chromatogram (<10 peaks with intensity >10ˆ7 counts s-1), unbiased retention index marker detection (MS similarity>800, validity of intensity range for high m/z marker ions), retention index calculation by fifth order polynomial regression. Spectra were cut to 5% base peak abundance and matched to database entries from most to least abundant spectra using the following matching filters: retention index window ±2,000 units (equivalent to about ±2 s retention time), validation of unique ions and apex masses (unique ion must be included in apexing masses and present at >3% of base peak abundance), mass spectrum similarity must fit criteria dependent on peak purity and signal/noise ratios and a final isomer filter. Failed spectra are automatically entered as new database entries if s/n > 25, purity <1.0, and presence in the biological study design class was >80%. All thresholds reflect settings for ChromaTOF 2.32. Quantification is reported as peak height for reasons noted above.

### Bioinformatics and statistical analysis

All data processing and statistical analyses used R v4.4.1 (https://www.R-project.org/) and Bioconductor v3.19 (21). Abundance values were read in using either readxl v1.4.3 (https://CRAN.R-project.org/package=readxl) or readr v2.1.5 (https://CRAN.R-project.org/package=readr), with feature id and sample id cleaning using janitor v2.2.0 (https://CRAN.R-project.org/package=janitor) and dplyr v1.1.4 (https://CRAN.R-project.org/package=dplyr). Plots were generated using either singly or in combination packages: ggplot2 v3.5.1 (22); geom_sina from ggforce v0.4.2 (https://CRAN.R-project.org/package=ggforce); patchwork v1.3.0 (https://CRAN.R-project.org/package=patchwork); ComplexHeatmap v2.20.0 (23). Analysis workflows were coordinated using targets v1.8.0 (24).

All four omics datasets (transcriptomics, biogenic amines, primary metabolism, lipidomics) were processed and analyzed separately as they were acquired in separate runs of instrumentation. Omics features were kept for further analysis if they were present (raw abundance ≥ 10) in 75% or more in either the tumor or adjacent nontumor samples (visualizationQualityControl v0.5.1) (https://github.com/moseleyBioinformaticsLab/visualizationQualityControl). All data sets were stored in R and manipulated as DESeq2 datasets (v1.44.0) (25). Sample normalization used estimateSizeFactors from DESeq2.

#### Quality Control (QC)

Sample - sample correlations were calculated using information-content-informed Kendall-tau (ICI-Kt, ICIKendallTau v1.2.2) (26). Outlier samples to be removed prior to differential analysis were based on scores calculated from the median ICI-Kt values within tumor and adjacent nontumor treatments and outliers detected using boxplot.stats. Outliers and final sample numbers across all analyses can be found in Supplemental Fig. S2 and Supplemental Table S1, respectively.

#### Principal Component Analysis (PCA)

Missing values were set to zero, and abundances were log-transformed using log(x + 1) (log1p function in R), and principal components calculated using the prcomp function, with centering and no scaling. Principal component variances and percentages of total were calculated using visqc_score_contributions.

#### Differential Comparisons

Patients who had either a tumor or an adjacent nontumor sample removed from outlier detection had the other sample removed prior to differential analysis. Any duplicate samples were collapsed using the collapseReplicates function from DESeq2. Statistics were calculated using DESeq2, with dispersions to fit the mean intensity using the parametric method for transcriptomics and the local method for the metabolomics datasets. The linear model for the differential analysis of tumor versus adjacent nontumor included the patient to account for the paired nature of the samples, specifically using the design of ∼ patient + treatment, and then extracting the contrast of treatment; tumor versus adjacent nontumor in the DESeq results. For the determination of differential features in tumor versus adjacent non-tumor between females and males, a secondary variable was created to handle the confounding of sex and patient (ind_rep, as noted in the DESeq2 vignette). The design used is ∼ sex + sex:treatment + sex:ind_rep, with a list contrast of “sexf.treatmentcancerous” and “sexm.treatmentcancerous” to extract the comparison of female(tumor - nontumor) - male(tumor - nontumor). *P*-values were adjusted using the Benjamini-Hochberg method (27).

#### Annotation Enrichment

Reactome pathway annotations for Ensembl genes were downloaded from the reactome pathway website on 2024-02-23 (28). Reactome pathway annotations for ChEBI metabolites were downloaded from the reactome pathway website. Metabolites were mapped to ChEBI by a combination of InChiKey’s generated by OpenBabel (v3.1.1) (29) from the download of ChEBI to InChI, as well as through mapping of KEGG compounds. Lipids were annotated with their lipid class, chain lengths, total chain lengths, and degree of unsaturation using custom code to parse the lipid IDs provided by West Coast Metabolomics.

Hypergeometric enrichment used all annotated features as the universe, and those features with an Padj ≤0.01 as the significant set, with features split by log-fold-change direction (positively and negatively changed), using the hypergeometric_feature_enrichment in categoryCompare2 v0.200.2 (30). Enrichment *P*-values were adjusted using the Benjamini-Hochberg method. Binomial enrichment used all features regardless of *P*-value, using the binomial_feature_enrichment function in categoryCompare2, with annotations that have at least six features, the null hypothesis that the ratio of positively to negatively changed features should be 0.5, and adjusting *P*-values using the Benjamini-Hochberg method. Lipid annotations tested included the lipid class, individual chain length, total chain length, degree of unsaturation, as well as various combinations of the annotations.

#### Spearman Correlation of Transcripts and Metabolites

Correlations of all metabolites were calculated with all of the transcripts across the samples common to all omics methods, using Spearman correlation, with zero representing a missing value. Significantly changed transcripts (p-adjusted ≤0.01) with significant correlations (p-adjusted ≤0.01) to either compounds (biogenic amines & primary metabolism) or lipids that are associated with a significant binomial enriched category and with log-fold-change in the same direction as the overall direction of the enrichment (pathway or lipid annotation, p-adjusted ≤0.05) were extracted. The Spearman correlation was used as input for calculating the Euclidean distances between the metabolites and the genes, and then clustered using hierarchical clustering via hclust, and ordered using dendsort v0.3.4 (https://github.com/evanbiederstedt/dendsort). Clusters of metabolites and transcripts were defined in two passes. The first was by splitting the hierarchical clustering into x clusters using cutree, defined by the number of clusters desired. Subsequently, chosen clusters were split into sub-clusters by pruning away branches not in the desired cluster (via the prune function of dendextend v1.18.0 (31)), and then splitting the remainder again into the number of desired clusters using cutree.

Hypergeometric enrichment of transcripts and metabolites in each cluster used all measured features as the universe, and the members of the cluster as the significant set. *P*-values were adjusted using the Benjamini-Hochberg method.

## Results

### Patient characteristics

**Fig. 1:**
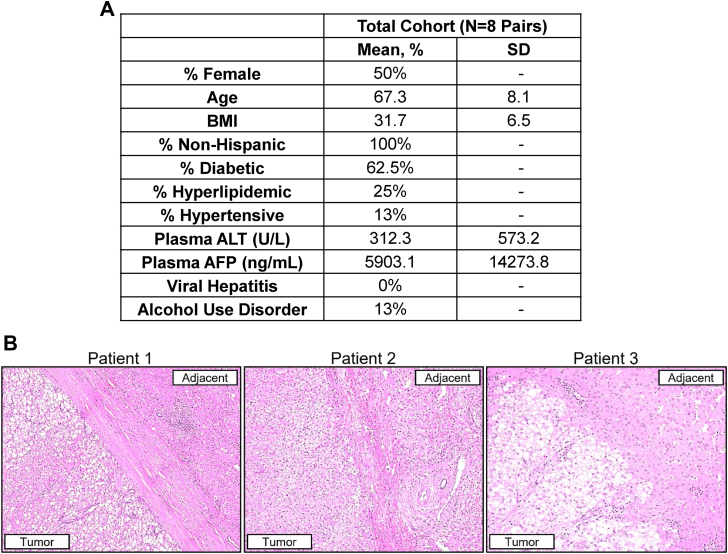
Patient Characteristics and Tumor Histology. A: Patient cohort demographics and clinical data. B: H&E stained liver sections from 3 patients highlighting tumor and adjacent nontumor tissue used in the analysis.

Eight human HCC tumors and adjacent paired non-tumor tissue were collected and used for analysis. All patients were non-Hispanic and consisted of four male and four female patients with a mean age of 67.3 years. Mean body mass index (BMI) was 31.7 kg/m^2^, with seven of the eight patients having a BMI greater than MASLD cardiometabolic criteria of 25 kg/m^2^. Five patients were diabetic or taking medications for diabetes, and two were hyperlipidemic or taking lipid-lowering therapies. Combined, seven of the eight patients met at least one cardiometabolic MASLD criterion, while one patient met criteria for MetALD due to a history of prior alcohol use disorder (∼140g of alcohol/day) (11). None of the patients had documented surface antigens or antibodies for either hepatitis B or C. Patient characteristics and available clinical data are summarized in Fig. 1A.

Hematoxylin and eosin-stained images of tumor and adjacent nontumor tissue were verified by an independent pathologist. Tumor tissues were enriched in lipid vacuoles with clear inflammatory foci present in both the tumor and the nontumor tissue (Fig. 1B). Another histological feature was the presence of a fibrotic capsule surrounding the tumor tissue, which is commonly observed in HCC (32). Taken together, this patient cohort resembles that of the metabolically-driven steatohepatitic variant of HCC.

### Complement cascade and scavenging pathways are uniquely repressed in HCC

**Fig. 2:**
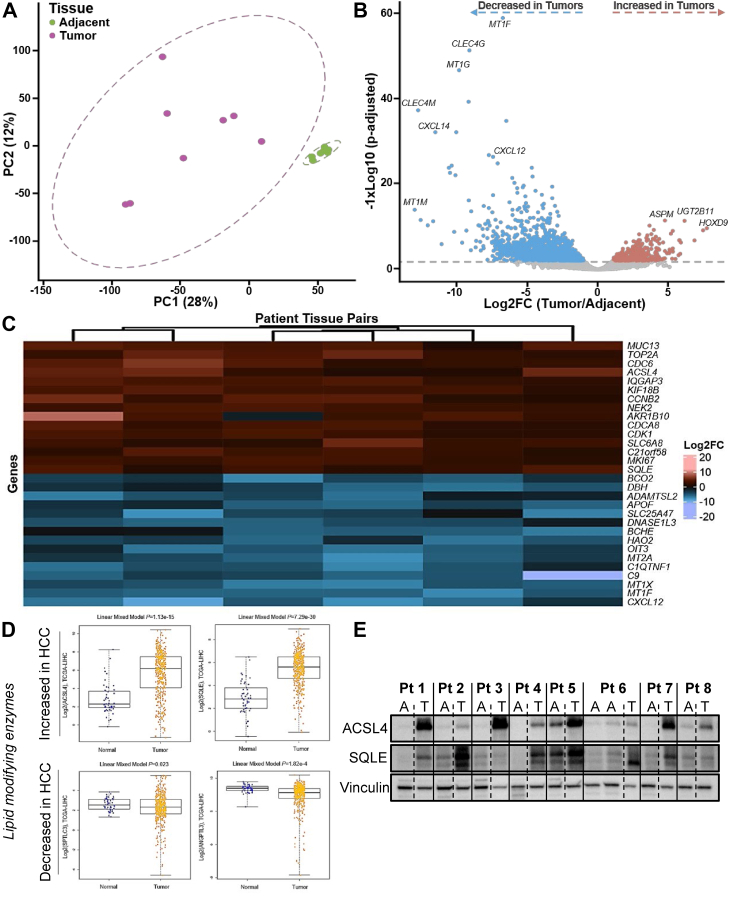
Lipid Metabolic Genes Exhibit Differential Abundance in Tumor and Adjacent Nontumor Tissue. A: Principal component analysis representing variation of transcripts across tumor (purple) and adjacent nontumor (green) tissue. B: Volcano plot highlighting genes which commonly increased (in red) or decreased (in blue) in tumors relative to nontumor tissue across all paired patient samples. C: A heatmap highlighting the top 15 increased (red/pink) and top 15 decreased (blue/purple) genes commonly identified across all paired samples (tumor/nontumor). D: Transcript levels of 4 lipid metabolic genes (*ACSL4, SQLE, SPTLC3, ANGPTL3*) in human normal liver versus HCC tumor tissue from TCGA-LIHC (n = 424). A fitted linear mixed model was used with resulting *P*-values corrected for multiple comparisons to control FDR. E: Immunoblotting of ACSL4 and SQLE from all paired patient samples (adjacent [A] vs. tumor [T]). Vinculin serves as loading control.

Bulk RNA-sequencing on paired tumor and adjacent nontumor tissue revealed 1,679 genes that met a pre-determined statistical cutoff of (p-adjusted value [Padj] ≤0.01). Of the 1679 genes identified, 1,398 (83.3%) were reduced while 281 (16.7%) were commonly increased in tumors across all paired patient samples. A list of differentially expressed genes (DEGs) can be found in Supplemental Table S2. Principal component analysis (PCA) revealed the transcriptome of tumors were profoundly different from that of adjacent nontumor tissues, with PC1 accounting for 28% and PC2 accounting for 12% of the variance (Fig. 2A). Given our patient cohort was 50% females, we inquired whether there were common DEGs in female tumor/adjacent versus that of male tumor/adjacent tissue. Tumor tissue, but not sex, was the major driver of transcriptomic differences via PCA (Supplemental Fig. S3A). Only 37 genes showed statistically significant (Padj < 0.01) differences in expression comparing female tumor/adjacent relative to male tumor/adjacent (Supplemental Fig. S3*B*, Supplemental Table S3). Notably, 10 of the 37 genes were common among all paired analyses (e.g. in the original 1,679 genes). The small subset of 27 genes show a distinct sexually dimorphic pattern of expression with 17 genes increased (*ABI3BP,* AC104809.2, B3GA*LT4, CCDC80, CCL18, CKB, FOXC1, GLIS2, HENMT1, IGHA2, MLF1, NPTX2, OXCT1, PPM1H, SESN3, SLC49A3, SMIM24*) and 10 genes decreased (*CLDN9, INHBA, PIK3C2G, SLC13A3, SLC22A3, SPINK1, STEAP1, TPRG1, U2AF1L5, UGT2A1*) in female tumor/adjacent versus male tumor/adjacent tissue (Supplemental Table S3). Overall, biological sex was not a major determinant of DEGs in HCC.

Consistent with prior studies (33, 34, 35), genes encoding DNA topoisomerase II alpha (*TOP2A*: Log2FC = 3.79; Padj = 5.44E-9), cell division cycle 6 (*CDC6*: Log2FC = 3.76; Padj = 3.51E-7), and marker of proliferation Ki-67 (*MKI67*: Log2FC = 2.93; Padj = 5.93E-9; Fig. 2B, C) were significantly increased across all paired tumors relative to nontumor tissue. Supporting gene enrichment analyses revealed pathways related to cell cycle control were elevated in tumors, with Padj ranging from 6.73E-13 for R-HSA-68886 “M Phase” to 6.55E-28 for R-HSA-1640170 “Cell Cycle” (Supplemental Table S4). Genes encoding proteins involved in metal chelation and scavenging of free radicals were largely repressed in tumor tissue (*MT1F, MT1X, MT2A;* Fig. 2B, C). Significant reductions in the 2-hydroxy acid oxidases (*HAO1*: Log2-FC = −2.31; Padj = 1.64E-5; *HAO2*: Log2-FC = −5.33; Padj = 6.21E-20) were also observed in tumor tissue (Fig. 2B, C), consistent with a previous report (36). The solute carrier family 25 member 47 (*SLC25A47*) gene encodes a liver-specific mitochondrial NAD + transporter that, when deleted, leads to impaired mitochondrial fatty acid oxidation, liver fibrosis, and HCC tumorigenesis in mice (37, 38). Consistent with its role in chronic liver disease, *SLC25A47* was significantly repressed in HCC across paired patient samples (Log2FC = −5.20; Padj = 2.63E-10). Further, genes involved in complement activation (*C9, C1QTNF1*) and lipoprotein metabolism (*APOF*) were commonly reduced in tumor tissue across all paired samples (Fig. 2B, C). Gene enrichment analyses revealed global reductions in complement signaling, scavenging receptors, and extracellular matrix organization pathways in HCC, specifically R-HSA-166658 “Complement cascade” with a Padj of 9.11E-19, R-HSA-2173782 “Binding and Uptake of Ligands by Scavenger Receptors” with Padj of 2.31E-16, and R-HSA-1474244 “Extracellular matrix organization” with Padj of 2.15E-11 (Supplemental Table S5).

We next set out to validate our bulk RNA-sequencing analysis by determining if the 15 most commonly up- and downregulated genes across paired tissues were also significantly altered in The Cancer Genome Atlas-Liver Hepatocellular Carcinoma (TCGA-LIHC). All 30 genes we identified in Figure 2C were significantly altered, and with similar directionality, across 424 human tumor and nontumor tissue samples from TCGA-LIHC (Supplemental Table S6). Notably, none of the top 30 DEGs exhibited sexually dimorphic expression across tumors, either via paired analyses (Supplemental Table S3) or from TCGA-LIHC (Supplemental Table S7). Of the most DEGs commonly across all paired tumor tissue (Fig. 2C; Supplemental Table S2), we identified 4 genes implicated in fatty acid, sterol, and ceramide metabolism that were independently validated via TCGA-LIHC. Acyl-CoA Synthetase long chain family member 4 (*ACSL4*; *P* = 1.13e-15) and squalene epoxidase (*SQLE*; *P* = 7.29e-30*)* transcript (Fig. 2D) and protein (Fig. 2E) levels were significantly more abundant in tumors, and their elevated expression associated with lower rates of overall survival (*ACSL4* Hazard Ratio [HR] = 1.356, Log-rank *P* = 0.0863; *SQLE* HR = 1.551, Log-rank *P* = 0.013) and 5-years survival (*ACSL4* HR = 1.416, Log-rank *P* = 0.0563; *SQLE* HR = 1.572, Log-rank *P* = 0.013) in patients with HCC (Supplemental Fig. S4A–D). This observation is consistent with independent reports showing both *ACSL4* (39, 40) and *SQLE* (41) accelerate HCC tumor growth in preclinical models. Meanwhile, serine palmitoyltransferase long chain base subunit 3 (*SPTLC3*; *P* = 0.023) and angiopoietin like 3 (*ANGPTL3*; *P* = 1.82e-4) transcript levels were significantly lower in tumors (Fig. 2D), and lower expression of *ANGPTL3* tended to associate with worse overall survival (HR = 0.776, Log-rank *P* = 0.148) and 5-years survival (HR = 0.733, Log-rank *P* = 0.088) in HCC patients, although these associations did not reach statistical significance (Supplemental Fig. S5A, B). *SPTLC3* transcript levels did not associate with overall prognosis in patients with HCC (Supplemental Fig. S5C, D).

### Ceramides and linoleic acid-enriched lipids are decreased in HCC

**Fig. 3:**
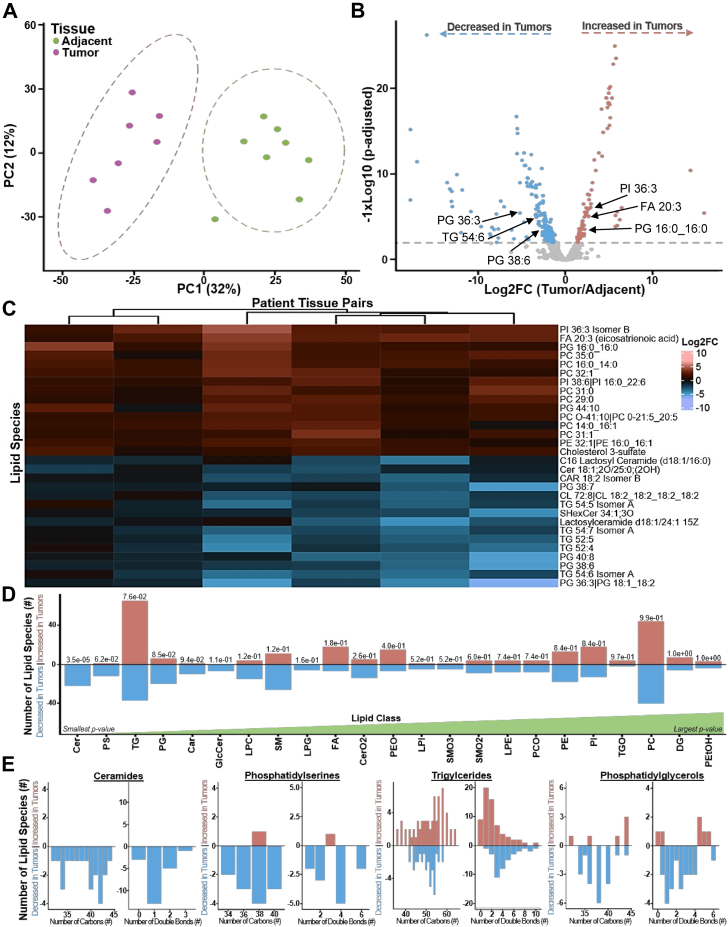
Ceramides, LA-enriched Lipids, and PS Are Decreased in Tumors. A: Principal component analysis of tumor (purple) and adjacent nontumor (green) tissue. B: Volcano plot highlighting specific lipids which commonly increased (in red) or decreased (in blue) in tumors relative to nontumor tissue across all paired patient samples. C: A heatmap highlighting the top 15 increased (red/pink) and top 15 decreased (blue/purple) lipids commonly identified across all paired samples (tumor/nontumor). D: Binomial analysis of lipid enrichment across 22 lipid classes (x-axis). The y-axis denotes the total number (#) of lipid species increased (red) or decreased (blue) in tumors relative to nontumor tissue across all paired analyses. Padj values increase from left to right across lipid classes. E: The number of carbons (left) and number of double bonds (right) in each of the top 4 lipid classes initially identified by binomial analysis in Figure 3D (ceramides, PS, triglycerides, and phosphatidylglycerols).

Given the profound lipid vacuole accumulation (Fig. 1B) and alterations in lipid metabolic genes in HCC (Fig. 2C–E), we next utilized LC-HRMS/MS to determine which lipid species were commonly altered in tumor versus adjacent nontumor tissue across all paired samples. PCA revealed the lipidome of tumors were profoundly different from that of adjacent nontumor tissue, with PC1 accounting for 32% and PC2 accounting for 12% of the variance (Fig. 3A). We initially detected a total of 2,238 lipid species, comprising of 547 annotated (24%) and 1,691 unannotated (76%) lipids. Of the original 2,238 lipid species detected, 292 met a pre-determined statistical cutoff of Padj ≤0.01. A majority were unannotated (250, or 86%) while 42 were previously annotated lipids (14%; Supplemental Table S8). Biological sex was not a major determinant of the tumor lipidome by PCA (Supplemental Fig. S6A), and no lipid species, annotated or unannotated, were significantly and distinctly altered in female tumor/adjacent versus male tumor/adjacent tissue (Supplemental Fig. S6B; Supplemental Table S9). The 15 most commonly increased lipids in HCC across all paired tissues, regardless of sex, were saturated and monounsaturated phospholipids, including PG16:0_16:0 (Log2FC = 2.03; Padj = 2.52E-4), PC35:0 (Log2FC = 1.97; Padj = 2.25E-4), PC16:0_14:0 (Log2FC = 1.91; Padj = 5.66E-6), and PC14:0_16:1 (Log2FC = 1.47; Padj = 8.01E-3; Fig. 3B, C; Supplemental Table S8). Meanwhile, of the top 15 most commonly decreased lipids in tumors, a majority were either ceramides or linoleic acid-containing (C18:2-LA) lipids, including reductions in C16 Lactosyl-ceramide (d18:1/16:0; Log2FC = −1.66; Padj = 5.61E-3), LA-enriched phospholipids (PG18:1_18:2; Log2FC = −3.36; Padj = 1.27E-6), LA-enriched cardiolipin (CL72:8: 18:2_18:2_18:2_18:2; Log2FC = −1.96; Padj = 4.34e-4), and LA-enriched acylcarnitine (CAR18:2; Log2FC = −1.85; Padj = 4.73E-4; Fig. 3B, C; Supplemental Table S8).

We next performed binomial enrichment analysis on the 547 annotated lipids to determine which lipid classes were most commonly and differentially affected across all paired tissue samples. Of the 23 lipid classes, ceramides were the only lipid class significantly different and decreased in HCC (Padj = 3.5E-5; Fig. 3D). Notably, all 22 ceramide lipids detected by LC-HRMS/MS, regardless of the number of carbons or degree of saturation, were decreased in tumors across all patient pairs (Fig. 3D, E). Similar to ceramides, phosphatidylserines (PS; Padj = 6.2E-2), phosphatidylglycerols (Padj = 8.5E-2), and acylcarnitines (Padj = 9.4E-2) all trended to decrease in HCC. On the contrary, triglycerides (Padj = 7.6E-2) and non-esterified fatty acids (NEFAs; Padj = 1.8E-1) trended to increase in tumors across all patient pairs. Of the total triglyceride pool, 100% of saturated triglycerides (Padj = 6.3E-2; class_total_db:TG:0 in Supplemental Table S10), 95% of MUFA-enriched triglycerides (Padj = 1.0E-3; class_total_db:TG:1 in Supplemental Table S10), and 92% of triglycerides with ≤ two double bonds were significantly increased in tumors relative to nontumor tissue. We observed similar increases in fatty acyl chain saturation in phosphatidylcholine (Supplemental Fig. S7A) and in NEFAs (Supplemental Fig. S7B) of tumor tissue, while their respective unsaturated counterparts tend to be decreased in HCC (Fig. 3E; Supplemental Fig. S7A, B). Taken together, and relative to adjacent nontumor tissue across all paired samples, human HCC exhibits a lipid signature that consists of: 1) statistically significant reductions in ceramides and LA-enriched lipids, 2) statistically significant accumulation of saturated- and monounsaturated NEFAs, triglycerides, and phosphatidylcholine, and 3) plausible reductions in PS.

### *SPTLC3* transcript levels strongly associate with ceramides in HCC

**Fig. 4:**
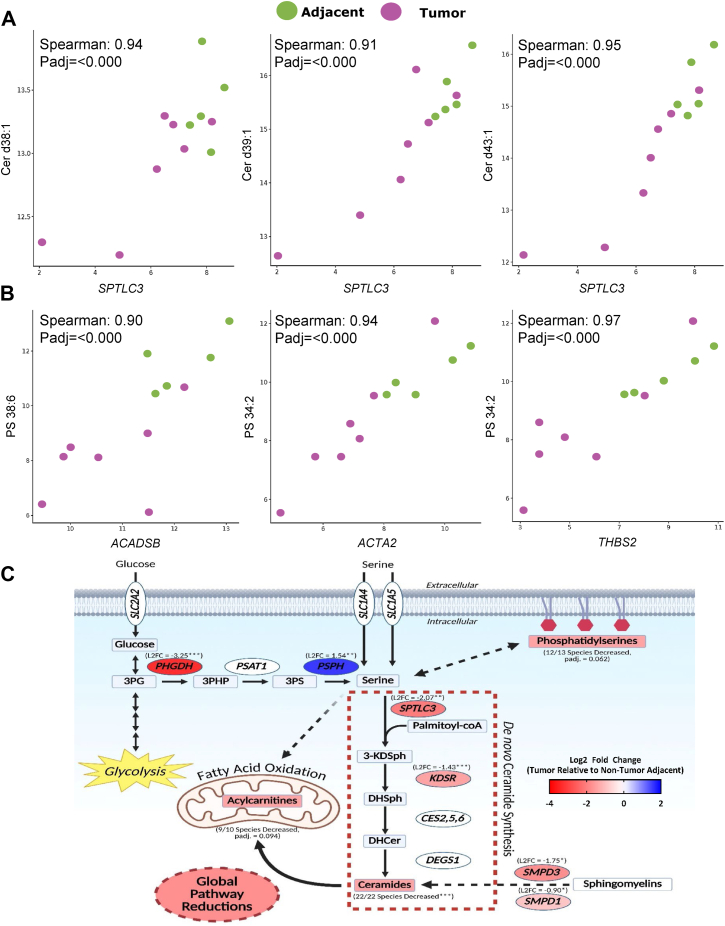
Transcripts Highly Associated With Ceramides and PS in HCC. A, B: Six highly significant associations between *SPTLC3*-ceramides (A) and gene-PS (B) across tumor (purple) and adjacent nontumor tissue (green). Spearman correlation coefficient and Padj values are provided in each panel. C: Graphical summary showing the interplay between serine availability, de novo ceramide synthesis, and mitochondrial fatty acid oxidation in HCC. The 3 lipid classes highlighted in red (PS, Acylcarnitines, Ceramides) were analyzed via binomial analysis with Padj values provided underneath each lipid class. Log2FCs of significantly changed transcripts are presented above their respective genes with colors corresponding to directionality and degree of change (red decreased; blue increased in HCC). Uncolored transcripts did not meet statistical cutoff. ∗Padj < 0.05, ∗∗Padj. < 0.01, ∗∗∗Padj. < 0.001.

Utilizing bulk RNA-sequencing and lipidomics platforms, we next sought to identify novel gene-lipid associations across paired tumor and adjacent nontumor tissue. Utilizing only significantly altered gene transcripts from bulk RNA-sequencing in Figure 2 and lipids associated with significantly enriched lipid categories in Figure 3, we employed Spearman correlation analyses between gene transcripts and lipid species. We initially identified a total of 5940 gene-lipid associations (Supplemental Fig. 8A; Supplemental Fig. 8A; Supplemental Tables S11 and S12), with 186 meeting both the predetermined statistical cutoff of Padj<0.01 and a Spearman’s rank correlation coefficient of | 0.90 | . Of the 186, 17 were negatively associated, while 169 were positively associated with specific lipid species (Supplemental Table S11). Given the significant reductions in total ceramides across all paired tumors (Fig. 3D, E), we sought to determine if any transcripts were strongly associated with ceramide species across tumor and adjacent nontumor tissue. We identified 35 genes that were significantly associated with at least one ceramide lipid species across all paired tissues (Supplemental Table S11). The rate-limiting enzyme of de novo ceramide synthesis, *SPTLC3*, was positively associated with 4 ceramide species: Cer d38:1 (Spearman: 0.94), Cer d39:1 (Spearman: 0.91), Cer d41:2 (Spearman: 0.90), and Cer d43:1 (Spearman: 0.95), with tumors exhibiting lower levels of both gene and lipid levels (Fig. 4A; Supplemental Table S11).

Endogenous synthesis of ceramides is regulated, in part, by free serine and palmitoyl-CoA availability; however, reports have shown that serine-derived from PS can ultimately be used as a substrate for ceramide synthesis (42). Intriguingly, ceramides and PS were the top 2 lipid classes decreased in HCC (Fig. 3D, E). We went on to identify an additional 12 genes that were significantly associated with at least one PS lipid species across all paired tissue. Notably, mitochondrial fatty acid oxidation and extracellular matrix proteins were the most prominently associated with PS lipids, including: *ACADSB* and PS38:6 (Spearman: 0.90), *TAGLN* and PS34:2 (Spearman: 0.91), *ACTA2* and PS34:2 (Spearman: 0.94), *THBS2* and PS34:2 (Spearman: 0.97), *ELN* and PS34:2 (Spearman: 0.92), and *MYH11* and PS34:2 (Spearman: 0.92; Fig. 4B; Supplemental Table S11).

To summarize our observations across paired patient samples in Figure 4C, genes involved in de novo serine (*PHGDH*) and ceramide (*SPTLC3, KDSR*) synthesis are significantly reduced in tumors. While *SPTLC3* transcript levels did not associate with overall HCC prognosis (Supplemental Fig. S5C, D), *PHGDH* was decreased in paired tumors (Log2FC = −3.25; Padj = 1.63E-6) and from TCGA-LIHC, with higher expression tending to be associated with worse overall survival (HR = 1.355, Log-rank *P* = 0.084) and 5-years survival (HR = 1.387, Log-rank *P* = 0.073; Supplemental Fig. S9A–C). These changes in transcript levels associated with global reductions in ceramides (22/22 lipids decreased in tumors; Fig. 3D, E; Padj = 3.46E-5), PS (12/13 lipids decreased in tumors; Fig. 3D, E; Padj = 0.06), and acylcarnitines (9/10 lipids decreased in tumors; Fig. 3D, E; Padj = 0.09). Taken together, the integration between lipidomic and transcriptomic platforms uncovers a link between serine availability, de novo ceramide synthesis, and mitochondrial fatty acid oxidation in human HCC (Fig. 4C). Functional validation and mechanistic exploration of these pathways in preclinical HCC models are warranted.

### Purine metabolites and acylcarnitines are selectively reduced in HCC

**Fig. 5:**
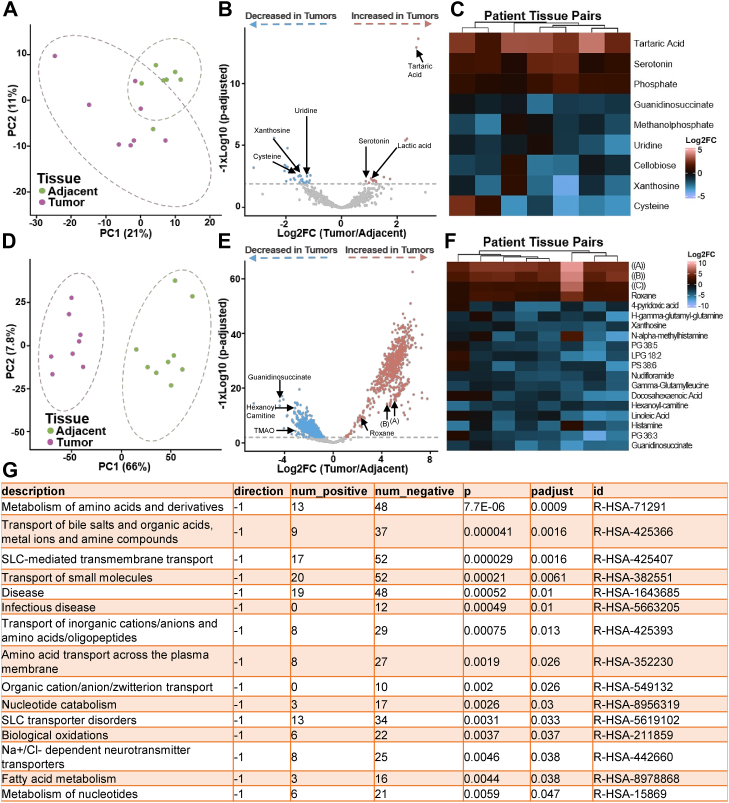
Purine Metabolites and Acylcarnitines Are Reduced in HCC. Metabolomics was performed on tumor and adjacent nontumor tissues utilizing the primary metabolism (A–C) and biogenic amine (D–F) platforms by West Coast Metabolomics. A, D: Principal component analysis from tumor (purple) and adjacent nontumor (green) tissue from metabolomic platforms. B, E: Volcano plot highlighting metabolites which commonly increased (in red) or decreased (in blue) in all paired tumors relative to nontumor tissue. C, F: Heatmaps highlighting the most increased (red/pink) and decreased (blue/purple) metabolites commonly identified across all paired samples (tumor/nontumor). G: Reactome pathway enrichment analyses highlighting the most significantly altered pathways across metabolomic datasets.

We next performed metabolomics utilizing two mass spectrometry-based platforms by West Coast Metabolomics. Using the primary metabolism platform (Fig. 5A–C), we detected a total of 775 metabolites (156 annotated; 619 unannotated) with only 47 metabolites meeting a predetermined statistical cutoff of Padj < 0.01. Of the 47, 9 (19.1%) have been previously annotated (Supplemental Table S8: PM in column R). Consistent with the low number of statistically significant metabolites, PCA analysis revealed an overlap between tumor and adjacent nontumor tissue with PC1 accounting for 21% and PC2 accounting 11% of the total variance across groups (Fig. 5A). When considering biological sex, only 1 unannotated primary metabolite (bin-base-id 34081) was identified as being significantly decreased (Log2FC = −3.80; Padj = 1.3E-03) in female tumor/nontumor tissue as compared to male tumor/nontumor tissue (Supplemental Fig. S10A, B; Supplemental Table S9). Of the top 10 most increased metabolites in tumors relative to nontumor tissue, a majority were related to cellular anabolic pathways including serotonin (Log2FC = 1.17; Padj = 6.00E-03), phosphate (Log2FC = 0.87; Padj = 7.50E-03), lactic acid (Log2FC = 0.67; Padj = 1.15E-02), phosphoethanolamine (Log2FC = 1.17; Padj = 2.93E-02), sucrose (Log2FC = 1.27; Padj = 4.10E-02), and hypoxanthine (Log2FC = 0.70; Padj = 9.94E-02). Meanwhile, end-products of purine metabolism were among the most decreased in tumors, including xanthosine (Log2FC = −1.90; Padj = 1.31E-03) and uric acid (Log2FC = −1.26; Padj = 1.53E-02).

Using the biogenic amine platform (Fig. 5D, E), we detected a total of 2,071 metabolites (426 annotated; 1,645 unannotated) with 1,649 being significantly altered (Padj < 0.01; 317 annotated metabolites) in tumors (Supplemental Table S8: Bioamines in column R). Given the significant number of altered metabolites, PCA analysis revealed biogenic amine profiles were profoundly different in tumors, with PC1 accounting for 66% and PC2 accounting for 7.8% of the variance (Fig. 5D). Biological sex was not a major determinant of biogenic amine metabolites between tumor and adjacent tissue (Supplemental Fig. S10C, D; Supplemental Table S9). Only 1 biogenic amine (4-hydroxybenzylcyanide) exhibited a sexually dimorphic pattern, with male tumors exhibiting greater abundance than female tumors (Log2FC = −4.70; Padj = 5.97E-3), relative to their respective paired tissue. The top 4 most increased metabolites across all tumors, independent of biological sex, were benzyldimethyltetradecylammonium cation ([A] in Fig. 5E, F; Log2FC = 5.17; Padj = 4.16E-16), N-benzyl-N,N-dimethyl-1-hexadecanaminium cation ([B] in Fig. 5E, F; Log2FC = 4.43; Padj = 6.84E-15), benzyldimethylstearylammonium cation ([C] in Fig. 5E, F; Log2FC = 2.61; Padj = 1.13E-07), and roxane (Log2FC = 2.41; Padj = 3.16E-10). Similar to what was observed with the primary metabolite platform, the most decreased metabolites across all tumors were related to purine and mitochondrial fatty acid metabolism, including: xanthosine (Log2FC = −2.91; Padj = 3.03E-12), xanthine (Log2FC = −1.70; Padj = 2.36E-4), uric acid (Log2FC = −2.21; Padj = 2.11E-09), adenosine (Log2FC = −2.65; Padj = 3.27E-07), inosine (Log2FC = −1.96; Padj = 3.08E-04), hexanoyl-carnitine (Log2FC = −3.03; Padj = 5.91E-15), palmitoyl-L-carnitine (Log2FC = −1.77; Padj = 8.23E-06), linoleic acid (Log2FC = −3.14; Padj = 6.25E-05), and docosahexaenoic acid (Log2FC = −3.01; Padj = 1.06E-06). Likewise, complimentary enrichment analysis revealed broad reductions in amino acid metabolism and transport (R-HSA-71291; Padj = 0.0009), transport of bile salts (R-HSA-425366; Padj = 0.0016) and small molecules (R-HSA-382551; Padj = 0.0061), and nucleotide (R-HSA-8956319; Padj = 0.03) and fatty acid metabolism (R-HSA-8978868; Padj = 0.038) in HCC tumors relative to nontumor tissue across all paired samples (Fig. 5G; Supplemental Table S13).

**Fig. 6:**
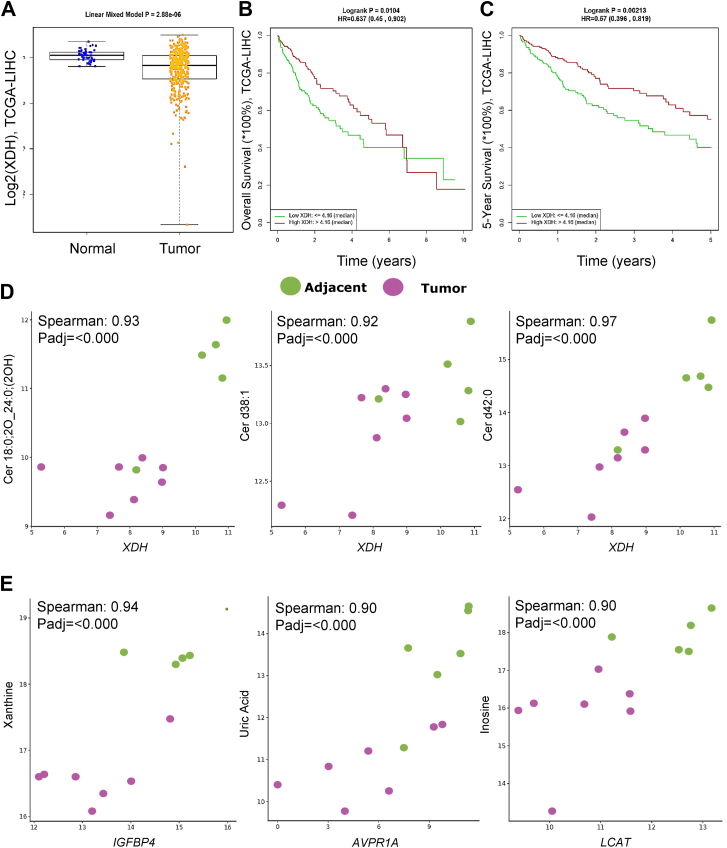
*XDH* Expression Associates with Overall Prognosis and Ceramides in HCC. A: Transcript levels of *XDH* in human normal liver versus HCC tumor tissue from TCGA-LIHC (n = 424). A fitted linear mixed model was used with resulting *P*-values corrected for multiple comparisons to control FDR. B, C: Kaplan-Meier curves associating median *XDH* transcript levels with overall survival (B) and 5-years survival (C) of HCC patients. Log-rank *P*-values and hazard ratios are provided. D, E: Six highly significant associations between *XDH*-ceramide lipids (D) and gene-purine metabolites (E) across tumor (purple) and adjacent nontumor tissue (green). Spearman correlation coefficient and Padj values are provided.

Integration of both metabolomic platforms revealed significant increases in hypoxanthine and reductions in xanthine, xanthosine, adenosine, inosine, and uric acid in paired tumor tissue (Fig. 5A–F). Two genes responsible for maintaining hypoxanthine/xanthine ratios are xanthine dehydrogenase (*XDH*) and hypoxanthine phosphoribosyl transferase 1 (*HPRT1*). *XDH* catalyzes serial oxidation of hypoxanthine-xanthine-uric acid, while *HPRT1* recycles hypoxanthine and guanine for inosine- and guanine monophosphate, respectively, as part of the purine salvage pathway. Previous independent reports have shown that *XDH* is low in HCC and other cancer types (43), while *HPRT1* is increased in non-HCC cancers (44). Consistent with these reports, we show that *XDH* is indeed lower in paired tumors (Log2FC = −2.35; Padj = 2.99E-7), and across 424 human samples from TCGA-LIHC with lower *XDH* expression associating with worse overall survival and 5-year survival (Fig. 6A–C). While low levels of *XDH* would be expected to increase the ratio of hypoxanthine-to-xanthine, *XDH* was also highly associated with 5 independent ceramide lipid species across paired tumor/nontumor tissue, including: Cer 18:0;2*O*_24:0;(2OH) (Spearman: 0.93), Cer d38:1 (Spearman: 0.92), Cer d42:0 (Spearman: 0.97), Cer d42:1 (Spearman: 0.91), and Cer-NP t42:0 (Spearman: 0.91; Fig. 6D). Meanwhile, transcript levels of *HPRT1* were significantly elevated in tumors from TCGA-LIHC, but expression did not associate with overall prognosis (overall survival: Log-rank *P* = 0.295; 5-years survival: Log-rank *P* = 0.32) in HCC patients (Supplemental Fig. S11A–C).

We then took an unbiased approach to identify all transcripts that commonly associated with purine metabolites across paired tissues. We initially identified a total of 2800 gene-metabolite associations (Supplemental Fig. S12; Supplemental Tables S14 and S15), with 117 that met the predetermined statistical cutoff of Padj<0.01 and a Spearman’s rank correlation coefficient of | 0.90 | . Of the 117, 11 genes (*IGFBP4, MT1G, LCAT, TDOD2, AVPR1A, IRF2BPL, RETREG1, GMNN, TMC8, TSPAN12, SEMA6D)* were identified as being highly associated with purine metabolites xanthine, inosine, and uric acid across tumor and nontumor tissue. Previous studies have shown that low levels of the insulin growth factor binding protein 4 (*IGFBP4*), vasopressin receptor 1A (*AVPR1A*), and lecithin cholesterol acyltransferase (*LCAT*) associate with poor prognosis in HCC patients (45, 46, 47). All of these genes were significantly reduced across paired tumors and significantly associated with xanthine (Spearman: 0.94), uric acid (Spearman: 0.90), and inosine (Spearman: 0.90), respectively. Taken together, our studies identify known (*XDH*) and unknown (*IGFBP4, AVPR1A, LCAT*) transcript-purine metabolite associations implicating these pathways in human HCC etiology.

## Discussion

The overall objective of this manuscript was to provide a comprehensive characterization of the transcriptome, lipidome, and metabolome of human steatohepatitic-driven HCC. Data integration across omics platforms revealed several notable observations in tumors relative to nontumor tissue: (1) ceramides, LA-enriched lipids, and PS were decreased; (2) fatty acyl chain saturation was increased; (3) genes involved in complement signaling, binding and uptake of ligands by scavenger receptors, and extracellular matrix pathways were decreased; (4) and metabolites involved in purine and fatty acid metabolism were decreased. Tumor tissue, but not biological sex, was the major determinant of DEGs and metabolites across paired analyses. Established, as well as novel, transcript-metabolite associations (117 gene-metabolite; 186 gene-lipid) were identified, thereby linking gene expression patterns to metabolic perturbations in HCC.

One of the most significant observations in human tumors was the reduction in total ceramide levels. Ceramides are bioactive lipids important for a variety of biological processes, including cancer cell growth, proliferation, and survival (48). A previous independent report demonstrated a 2-fold reduction in all ceramide lipids in HCC tissue relative to nontumor tissue (49). Consistent with this report, we also report a ∼3-fold reduction across all 22 ceramide lipid species regardless of acyl chain length or saturation. While marked reductions in total ceramide levels in HCC have been observed herein and by others (49), the association of serum ceramides with HCC appears to depend on chain length and degree of saturation. For instance, plasma CER(d18:1/20:1) has been shown to positively associate with tumor burden, inflammation, and shorter recurrence-free survival (RFS), while CER(d18:1/22:1) was associated with longer RFS (50). Other reports have demonstrated that all ceramide species accumulate in patients with cirrhosis and HCC relative to those without HCC (51). The mechanism(s) by which tissue and serum ceramide levels differ across HCC etiologies and disease stage is unknown and warrants further investigation.

We initially set to determine the mechanism(s) driving reductions in tumor ceramide levels. RNA-sequencing revealed significant reductions in ceramide de novo biosynthetic enzymes, *SPTLC3* and *KDSR*, as well as the sphingomyelin phosphodiesterase enzymes, *SPMD3* and *SMPD1*. Given sphingomyelins were largely unchanged, we reasoned that de novo synthesis of ceramides were likely reduced in tumors. Endogenous synthesis of ceramides is driven by free serine and palmitoyl-CoA availability. Intriguingly, both free- and PS-derived serine can be used as a substrate for ceramide synthesis (42), and PS was the second most decreased lipid class (behind ceramides) in tumors across all paired tissues. However, the endogenous synthesis of free serine is also likely reduced in tumor tissue due to the large and statistically significant reduction in the rate-limiting enzyme *PHGDH* and concomitant reductions in free serine levels via the biogenic amine platform. Pharmacological inhibition of *PHGDH* and serine deprivation have been shown to suppress oxidative metabolism, induce mitochondrial fragmentation, reduce acylcarnitines and ceramides, and exacerbate mitochondrial-driven diseases (52, 53). Notably, in colon cancer cells, ceramide supplementation can partially restore the mitochondrial defects observed with serine deprivation (52). Taken together, our paired analyses suggests that reductions in serine availability may lead to lower de novo ceramide synthesis and impaired mitochondrial fatty acid oxidation in steatohepatitic-driven HCC.

Genetic loss-of-function variants in *ANGPTL3* are significantly associated with reduced cardiovascular disease (54), and antibody-mediated inhibition of *ANGPTL3* has been shown to reduce apoB-containing lipoproteins in mice and humans (54, 55). However, conflicting reports have shown liver-specific antisense oligonucleotide-mediated inhibition of *ANGPTL3* leads to increased hepatotoxicity (via ALT and AST levels) and increased hepatic fat content (56, 57), while RNA interference targeting *ANGPTL3* decreased atherogenic lipoproteins but did not increase liver fat (58). In our analysis, human steatohepatitic tumors exhibited significantly lower *ANGPTL3* transcript levels as compared to paired nontumor tissue, which was further validated across 424 human samples in TCGA-LIHC. The reductions in *ANGPTL3* transcript levels tended to associate with prognosis, as low *ANGPTL3* associated with reduced overall survival and 5-years survival in HCC patients. Independent and supporting studies have confirmed lower *ANGPTL3* expression in HCC tissues, and forced ectopic expression of *ANGPTL3* re-sensitized sorafenib-resistant cells to sorafenib via modulation of carnitine palmitoyltransferase 1A-mediated fatty acid oxidation (59). Conflicting *in vitro* studies, however, have shown that CRISPR-mediated deletion of *ANGPTL3* in HepG2 cells reduced apoB100 secretion and promoted fatty acid oxidation (60), while downregulation of *ANGPTL3* via siRNA in human hepatocytes increased neutral lipid accumulation via reduced fatty acid oxidation (61). Consistent with a role of *ANGPTL3* in mediating mitochondrial metabolism, we identified a strong positive association with *ANGPTL3* transcripts and LA-enriched phospholipids, including PC16:0_18:2, PC34:2, and PE34:2. Linoleic acid is the most abundant FA in the cardiolipin pool (62) and LA-enrichment into cardiolipin and other phospholipids (such as PE) associate with mitochondria uncoupling, oxygen consumption rate, and improve mitochondrial respiration (63). A reduction of C18:2-cardiolipin has been observed throughout disease progression from MASLD to MASH (62). While *ANGPTL3* did not associate with C18:2-cardiolipin in our studies, a significant 3.9-fold reduction in LA-enriched cardiolipin (CL72:8; 4-LA acyl chains) was observed in HCC tumors which coincided with broad reductions in tumor acylcarnitine levels. A mechanistic understanding of how *ANGPTL3* modulates LA and mitochondrial metabolism, particularly in HCC, is warranted. Therapeutic approaches to target circulating versus hepatic ANGPTL3 need to be carefully considered when assessing CVD versus HCC risk reduction.

Lipid saturation has been shown to associate with liver disease severity (64, 65, 66, 67, 68). Patients with MASLD exhibited reductions in PUFA-triglycerides (64), and stepwise reductions in PUFA-enriched triglycerides were observed in livers from healthy controls to patients with MASH (68). Conversely, liver triglyceride saturation is increased and positively associated with increasing proton density fat-fraction in adults with MASLD (65, 67). The selective increase in saturated and MUFA-enriched triglycerides, phosphatidylcholine, and NEFAs were observed in tumor relative to nontumor tissue. This is consistent with a previous report using mass spectrometry imaging techniques to show MUFA-enriched phospholipids accumulate in mouse and human HCC tumors, and associate with mouse models of accelerated hepatocyte proliferation (66). Complementary to the study by Hall *et al.* (66), we also observed an association of tumor MUFAs with elevations in stearoyl-CoA desaturase (SCD) transcript levels, as well as reductions in fatty acid oxidative pathways in tumors versus nontumor tissue. The link between reductions in fatty acid catabolism and MUFA accumulation have been further supported by our group, showing loss of CPT1a-mediated FAO in the liver promotes accumulation of MUFAs at the expense of DHA-containing PUFAs, despite reduced levels of SCD1 (13). The accumulation of MUFAs in tumors are likely explained by preferential utilization of glycolytic metabolism and accelerated de novo lipogenesis, which then, in turn, represses CPT1a-mediated FAO to collectively promote MUFA accumulation for hepatocyte proliferation and tumor growth. Additional studies on perturbations in fatty acid metabolism in this context are warranted.

Alterations in purine metabolites and broad reductions in acylcarnitines were commonly observed in paired tumors across metabolomic platforms. In particular, the increase in hypoxanthine with reductions in xanthine, xanthosine, adenosine, inosine, and uric acid are of interest. These changes are associated with significant reductions in *XDH* transcript levels in tumors from paired analyses, in TCGA-LIHC, and from previous reports (43, 69). Mechanistically, *XDH* transcript levels inversely associate with epithelial-mesenchymal transition scores, and loss of *XDH* promotes the migration and invasion of HCC cancer cells in a TGFβ-SMAD-dependent manner (69). Mice with global deletion of *Xdh* (XOR^−/−^) exhibit renal failure due to the accumulation of hypoxanthine and its inhibitory effect on nicotinamide mononucleotide, a precursor of NAD^+^ (70). Further, hypouricemia in *Xdh* KO mice results in a failure to thrive, impaired oxidative phosphorylation, and significant inflammation in the kidney (71). Thus, strategies to lower intracellular hypoxanthine and concurrently boost NAD^+^ levels in HCC tumors may be an effective therapeutic approach to alleviate tumor growth (72, 73).

It is important to acknowledge limitations in our analyses. The primary limitation is the small sample size. Given significant advancements in imaging modalities and noninvasive treatment options, HCC tissue resection is now only recommended in very early stage (Barcelona-Clinic Liver Cancer Staging System: BCLC-0) and in single intrahepatic early stage (BCLC-A) disease (74), thereby limiting the number of tissues available for research purposes. Despite the small sample size, a major strength of this article is the paired nature of the samples. This approach enables us to identify transcripts and metabolites that commonly increase or decrease across all pairwise comparisons, thereby strengthening the conclusions of the data across individual omic platforms. One technical limitation to the approach, however, is the use of bulk RNA-sequencing for transcriptomic profiling. The HCC tumor microenvironment is highly heterogeneous (i.e., malignant HCC cells, nonmalignant HCC cells, endothelial cells, fibroblasts, and immune cells); thus, the bulk approach limits our understanding of cell-specific transcriptional networks across the dataset. With the advancement in single-cell and spatial technology, future studies will allow us to interrogate these relationships at much higher resolution utilizing a combination of scRNA-seq and/or spatial transcriptomics integrated with MALDI-MSI approaches. Regardless, functional studies need to be performed to assess whether these gene-metabolite relationships identified have a causal role in disease progression. Another limitation in our analysis is the amount of clinical data available on this patient cohort. Limited information regarding the stage of disease, cancer treatment(s), disease etiology, and pathological reports were available. Future studies stratifying these types of omics datasets across disease stage and/or etiology (viral vs. non-viral sources), for example, would allow for better identification of novel drivers of disease progression.

In conclusion, this manuscript was focused on describing transcript-metabolite associations in steatohepatitic-driven HCC, and we hope this research will serve as a valuable resource to other HCC researchers and lead to new pharmacological targets that may be exploited for therapeutic benefit.

## Data availability

All processed data, data analyses, and analysis code have been deposited in Zenodo: https://dx.doi.org/10.5281/zenodo.18227400. RNA-sequencing data have been deposited in GEO with the accession number GSE320365. Lipidomics and metabolomics data were deposited at the NIH Common Fund’s National Metabolomics Data Repository, the Metabolomics Workbench (75), where it has been assigned a study ID ST004733. The project ID is PR003001, with analysis IDs of AN008002, AN008003, AN008004, AN008005, and AN008006. The DOI is https://dx.doi.org/10.21228/M8RS0N. Additional information and requests for resources and reagents should be directed to and fulfilled by the Lead Contact, Robert N. Helsley (robert.helsley@uky.edu).

## Supplemental data

This article contains supplemental data.

## Conflict of interest

The authors declare that they have no conflicts of interest with the contents of this article.

## Supplemental data

Supplemental Table S1 Human samples analyzed across omics platforms.

Supplemental Table S2 Differentially expressed genes in tumor versus adjacent nontumor tissue.

Supplemental Table S3 Differentially expressed genes in female tumor/nontumor versus male tumor/nontumor.

Supplemental Table S4 Gene pathway enrichment analysis increased in tumors.

Supplemental Table S5 Gene pathway enrichment analysis decreased in tumors.

Supplemental Table S6 TCGA-LIHC validation of top 30 DEGs from paired analyses.

Supplemental Table S7 TCGA-LIHC sex-tumor interaction for top 30 DEGs from paired analyses.

Supplemental Table S8 Differential lipid and metabolite abundance in tumor versus adjacent nontumor tissue.

Supplemental Table S9 Differential lipid and metabolite abundance in female tumor/nontumor versus male tumor/nontumor.

Supplemental Table S10 Binomial lipid enrichment analysis.

Supplemental Table S11 Gene-lipid correlations in tumor versus adjacent nontumor tissue.

Supplemental Table S12 Hypergeometric enrichments of gene-lipid correlations.

Supplemental Table S13 Binomial metabolite enrichment analysis.

Supplemental Table S14 Gene-metabolite correlations in tumor versus adjacent nontumor tissue.

Supplemental Table S15 Hypergeometric enrichments of gene-metabolite correlations.

Supplemental Figures Supplemental Figure 1. Experimental design. (A) An overview of the experimental design implemented in this manuscript. Image created by biorender.com. Supplemental Figure 2. Outlier detection across omics platforms. (A-D) Sina plots of sample median ICI-Kt correlations across the following platforms: (A) RNA-sequencing, (B) lipidomics, (C) primary metabolism, and (D) biogenic amines and small molecule metabolites. Color indicates disease status (purple=tumor; green=nontumor) and shape indicates outlier status (triangle=outlier). Supplemental Figure 3. Biological sex is not a major driver of DEGs in HCC. (A) Principal component analysis representing variation of transcripts across biological sex (female – pink; male – blue) and tumor (purple) and adjacent nontumor (green) tissue. (B) UpSet plot comparing the number of significantly different transcripts for sex and tumor. Supplemental Figure 4. *ACSL4* and *SQLE* levels associate with HCC prognosis. (A-D) Kaplan-Meier curves associating median *ACSL4* and *SQLE* transcript levels with overall survival (A, C) and 5-year survival (B, D) of HCC patients. Data are pulled from TCGA-LIHC with log-rank *P*-values and hazard ratios provided. Supplemental Figure 5. *ANGPTL3* levels associate with HCC prognosis. (A-D) Kaplan-Meier curves associating median *ANGPTL3* and *SPTLC3* transcript levels with overall survival (A, C) and 5-year survival (B, D) of HCC patients. Data are pulled from TCGA-LIHC with log-rank *P*-values and hazard ratios provided. Supplemental Figure 6. Biological sex is not a major driver of lipid abundance in HCC. (A) Principal component analysis representing variation of lipids across biological sex (female – pink; male – blue) and tumor (purple) and adjacent nontumor (green) tissue. (B) UpSet plot comparing the number of significantly different lipids for sex and tumor. Supplemental Figure 7. Binomial lipid enrichment of phosphatidylcholine and NEFAs. (A) Total number of phosphatidylcholine lipids as a function of the total number of carbons (x-axis; left) or the total number of double bonds (x-axis; right). (B) The total number of NEFAs as a function of the total number of carbons (x-axis; left) or the total number of double bonds (x-axis; right). Number of lipids increased (in red) or decreased (in blue) in tumors relative to nontumor tissue across all paired analyses. NEFAs, nonesterified fatty acids. Supplemental Figure 8. Hypergeometric enrichments of gene-lipid correlations. (A) An enlarged heatmap of all 5941 gene-lipid associations. Hypergeometric enrichment shows clustering of extracellular matrix genes (transcriptomic cluster 1) with ceramide lipids (lipidomic cluster 2). Spearman correlation coefficient range from -1 (blue) to +1 (brown). X-axis, lipid clustering (i.e. metabolite); Y-axis, gene clustering. Supplemental Figure 9. *PHGDH* levels are decreased in HCC and associate with overall prognosis. (A) *PHGDH* transcript levels in human normal liver versus HCC tumor tissue from TCGA-LIHC (n=424). A fitted linear mixed model was used with resulting *P*-values corrected for multiple comparisons to control FDR. (B, C) Kaplan-Meier curves associating median *PHGDH* transcript levels with overall survival (B) and 5-year survival (C) of HCC patients. Data are pulled from TCGA-LIHC with log-rank *P*-values and hazard ratios provided. Supplemental Figure 10. Biological sex is not a major determinant of metabolite abundance in HCC. (A, C) Principal component analysis representing variation of primary metabolites (A) and biogenic amines and small molecules (C) across biological sex (female – pink; male – blue) and tumor (purple) and adjacent nontumor (green) tissue. (B, D) UpSet plot comparing the number of significantly different primary metabolites (B) and biogenic amines and small molecules (D) for sex and tumor. Supplemental Figure 11. *HPRT* levels are increased in tumors but do not associate with the survival of HCC Patients. (A) *HPRT* transcript levels in human normal liver versus HCC tumor tissue from TCGA-LIHC (n=424). A fitted linear mixed model was used with resulting *P*-values corrected for multiple comparisons to control FDR. (B, C) Kaplan-Meier curves associating median *HPRT* transcript levels with overall survival (B) and 5-year survival (C) of HCC patients. Data are pulled from TCGA-LIHC with log-rank *P*-values and hazard ratios provided. Supplemental Figure 12. Hypergeometric enrichments of gene-metabolite correlations. (A, B) An enlarged heatmap of all 2800 gene-metabolite associations. (A) Hypergeometric enrichment shows clustering of extracellular matrix genes (transcriptomic cluster 2) with nucleotide repair and Akt signaling pathways (metabolites cluster 1). (B) Hypergeometric enrichment shows clustering of fatty acid metabolic genes (transcriptomic cluster 1) with nucleotide repair and Akt signaling pathways (metabolites cluster 1). Spearman correlation coefficient range from -1 (blue) to +1 (brown). X-axis, metabolite clustering (i.e. biogenic amines + primary metabolites); Y-axis, gene clustering. Supplemental Figure 13. Uncropped western blot images. Uncropped western blot images from Fig. 2E with antibody and acquisition information provided.
