# Supplementary material for: Integrated multi-omic analyses reveal novel gene–metabolite relationships in human steatohepatitic hepatocellular carcinoma
Source: J Lipid Res. 2026 Jun 17;67(7):101081. doi: 10.1016/j.jlr.2026.101081 (PMC13400273; doi:10.1016/j.jlr.2026.101081)
Supplement: Supplemental Figures — Supplemental Figure 1. Experimental design. (A) An overview of the experimental design implemented in this manuscript. Image created by biorender.com. Supplemental Figure 2. Outlier detection across omics platforms. (A-D) Sina plots of sample median ICI-Kt correlations across the following platforms: (A) RNA-sequencing, (B) lipidomics, (C) primary metabolism, and (D) biogenic amines and small molecule metabolites. Color indicates disease status (purple=tumor; green=nontumor) and shape indicates outlier status (triangle=outlier). Supplemental Figure 3. Biological sex is not a major driver of DEGs in HCC. (A) Principal component analysis representing variation of transcripts across biological sex (female – pink; male – blue) and tumor (purple) and adjacent nontumor (green) tissue. (B) UpSet plot comparing the number of significantly different transcripts for sex and tumor. Supplemental Figure 4. ACSL4 and SQLE levels associate with HCC prognosis. (A-D) Kaplan-Meier curves associating median ACSL4 and SQLE transcript levels with overall survival (A, C) and 5-year survival (B, D) of HCC patients. Data are pulled from TCGA-LIHC with log-rank P-values and hazard ratios provided. Supplemental Figure 5. ANGPTL3 levels associate with HCC prognosis. (A-D) Kaplan-Meier curves associating median ANGPTL3 and SPTLC3 transcript levels with overall survival (A, C) and 5-year survival (B, D) of HCC patients. Data are pulled from TCGA-LIHC with log-rank P-values and hazard ratios provided. Supplemental Figure 6. Biological sex is not a major driver of lipid abundance in HCC. (A) Principal component analysis representing variation of lipids across biological sex (female – pink; male – blue) and tumor (purple) and adjacent nontumor (green) tissue. (B) UpSet plot comparing the number of significantly different lipids for sex and tumor. Supplemental Figure 7. Binomial lipid enrichment of phosphatidylcholine and NEFAs. (A) Total number of phosphatidylcholine lipids as a function of the to [file mmc16.pptx]

## Slide 1
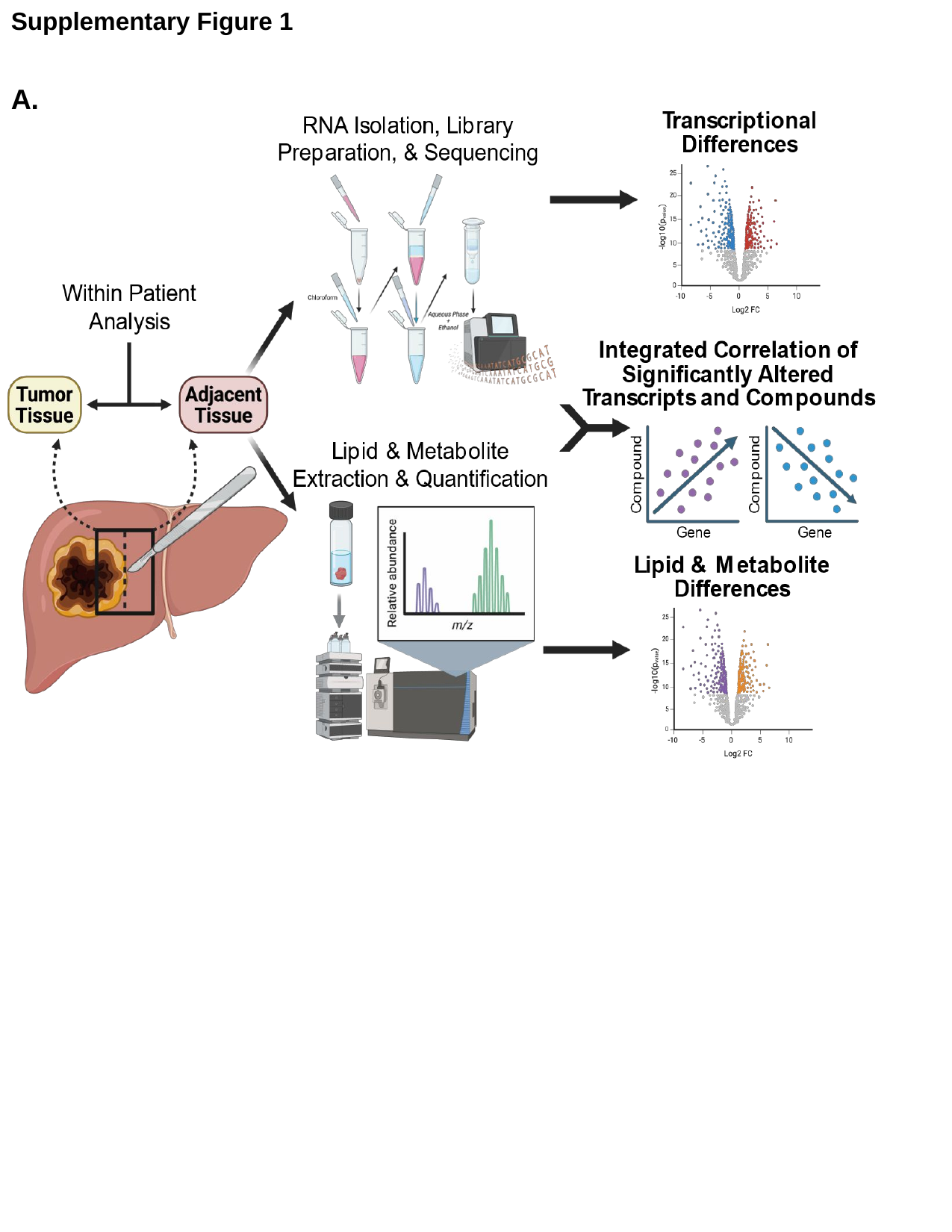

Supplementary Figure 1
A.

## Slide 2
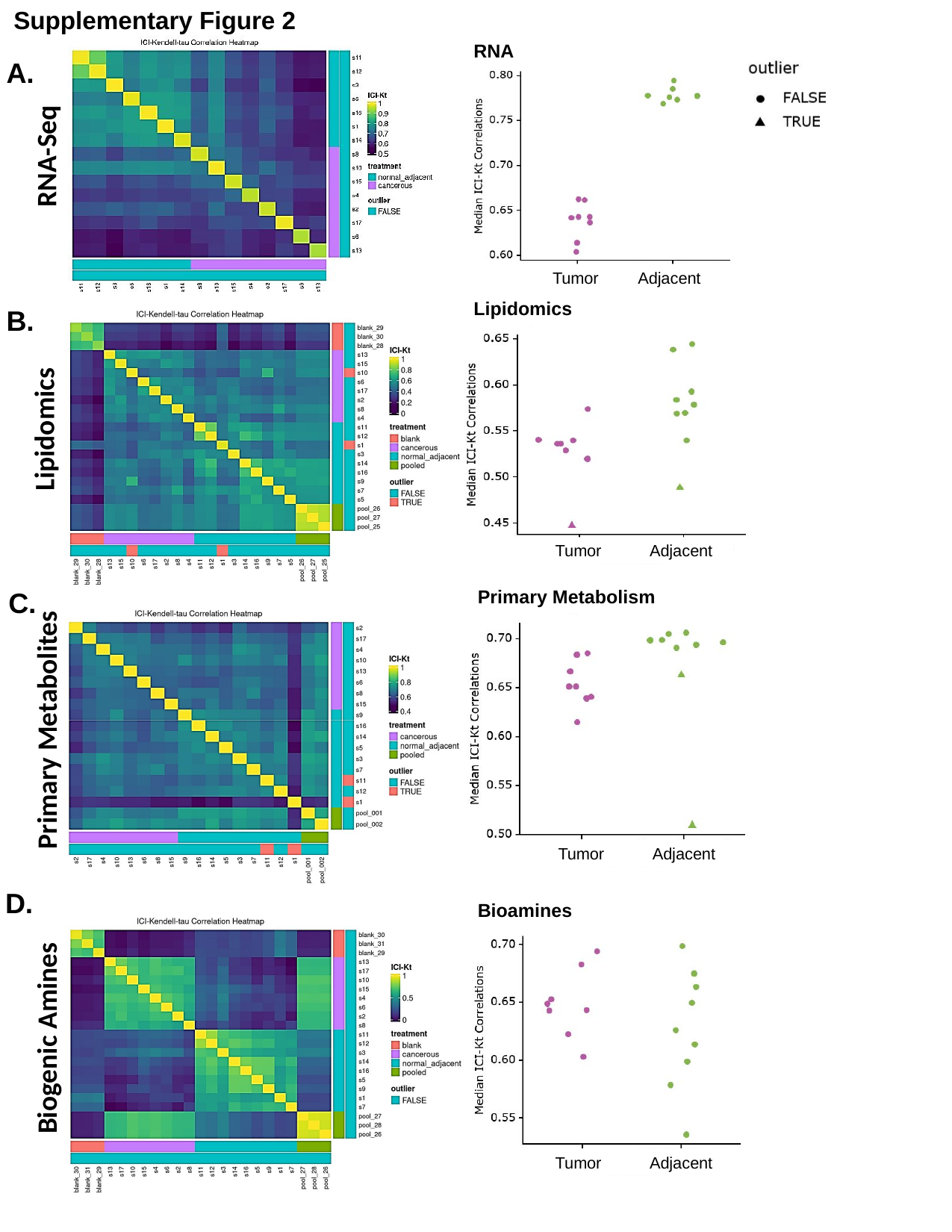

Supplementary Figure 2
RNA
A.
RNA-Seq
 Tumor Adjacent
Lipidomics
B.
Lipidomics
 Tumor Adjacent
C.
Primary Metabolism
Primary Metabolites
 Tumor Adjacent
D.
Bioamines
Biogenic Amines
 Tumor Adjacent

## Slide 3
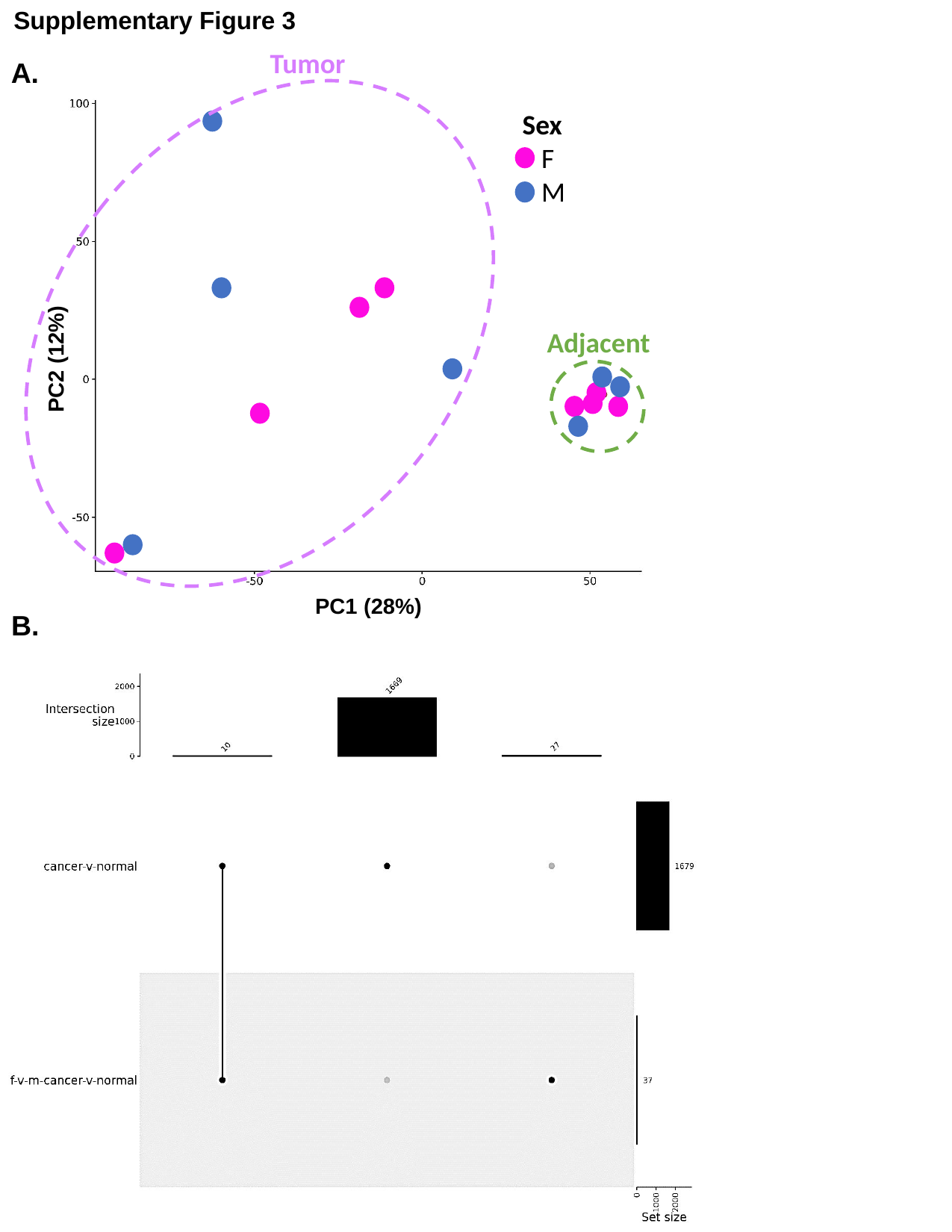

Supplementary Figure 3
Tumor
A.
 Sex
 F
 M
Adjacent
PC2 (12%)
PC1 (28%)
B.

## Slide 4
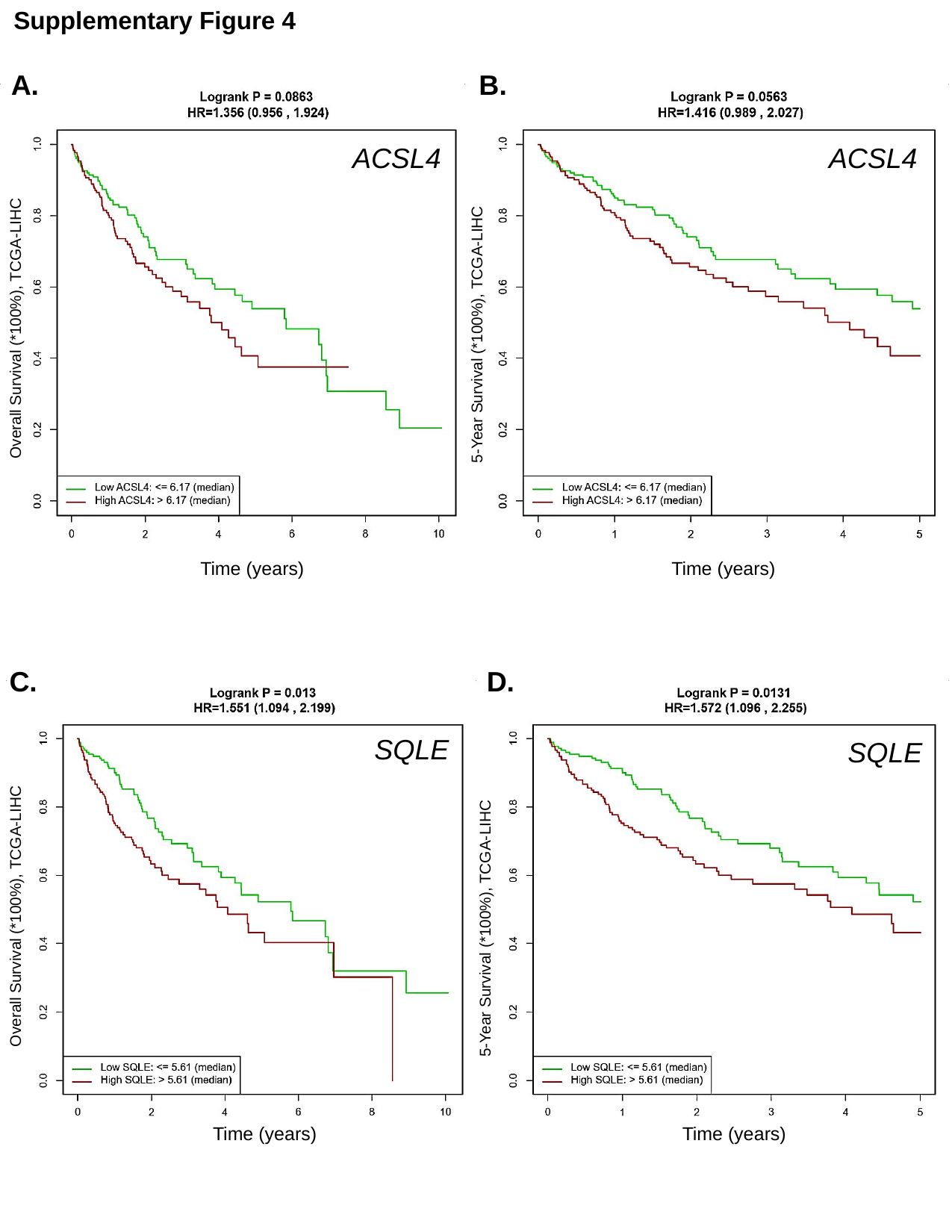

Supplementary Figure 4
A.
B.
ACSL4
ACSL4
 Overall Survival (*100%), TCGA-LIHC
 5-Year Survival (*100%), TCGA-LIHC
 Time (years)
 Time (years)
C.
D.
SQLE
SQLE
 Overall Survival (*100%), TCGA-LIHC
 5-Year Survival (*100%), TCGA-LIHC
 Time (years)
 Time (years)

## Slide 5
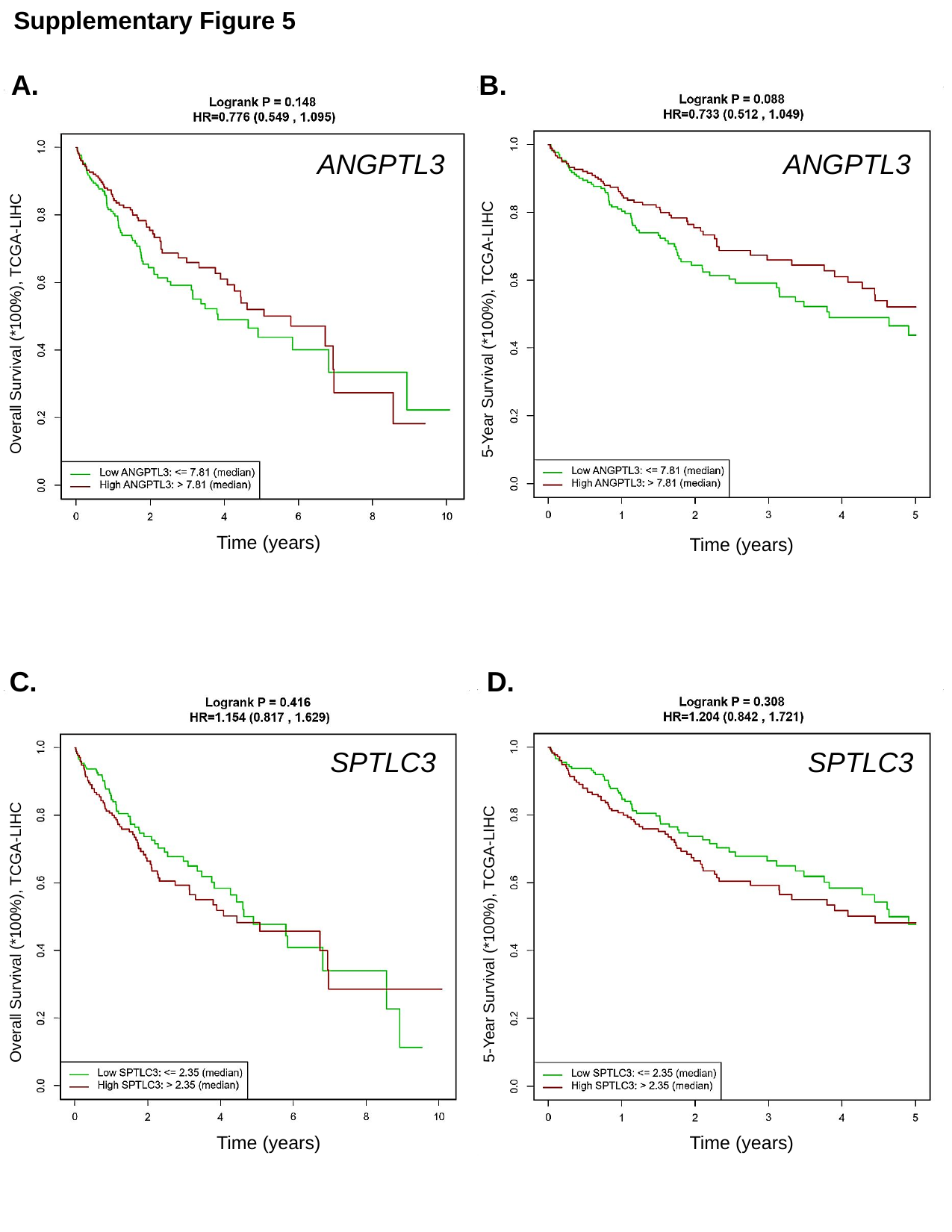

Supplementary Figure 5
A.
B.
ANGPTL3
ANGPTL3
 Overall Survival (*100%), TCGA-LIHC
 5-Year Survival (*100%), TCGA-LIHC
 Time (years)
 Time (years)
C.
D.
SPTLC3
SPTLC3
 5-Year Survival (*100%), TCGA-LIHC
 Overall Survival (*100%), TCGA-LIHC
 Time (years)
 Time (years)

## Slide 6
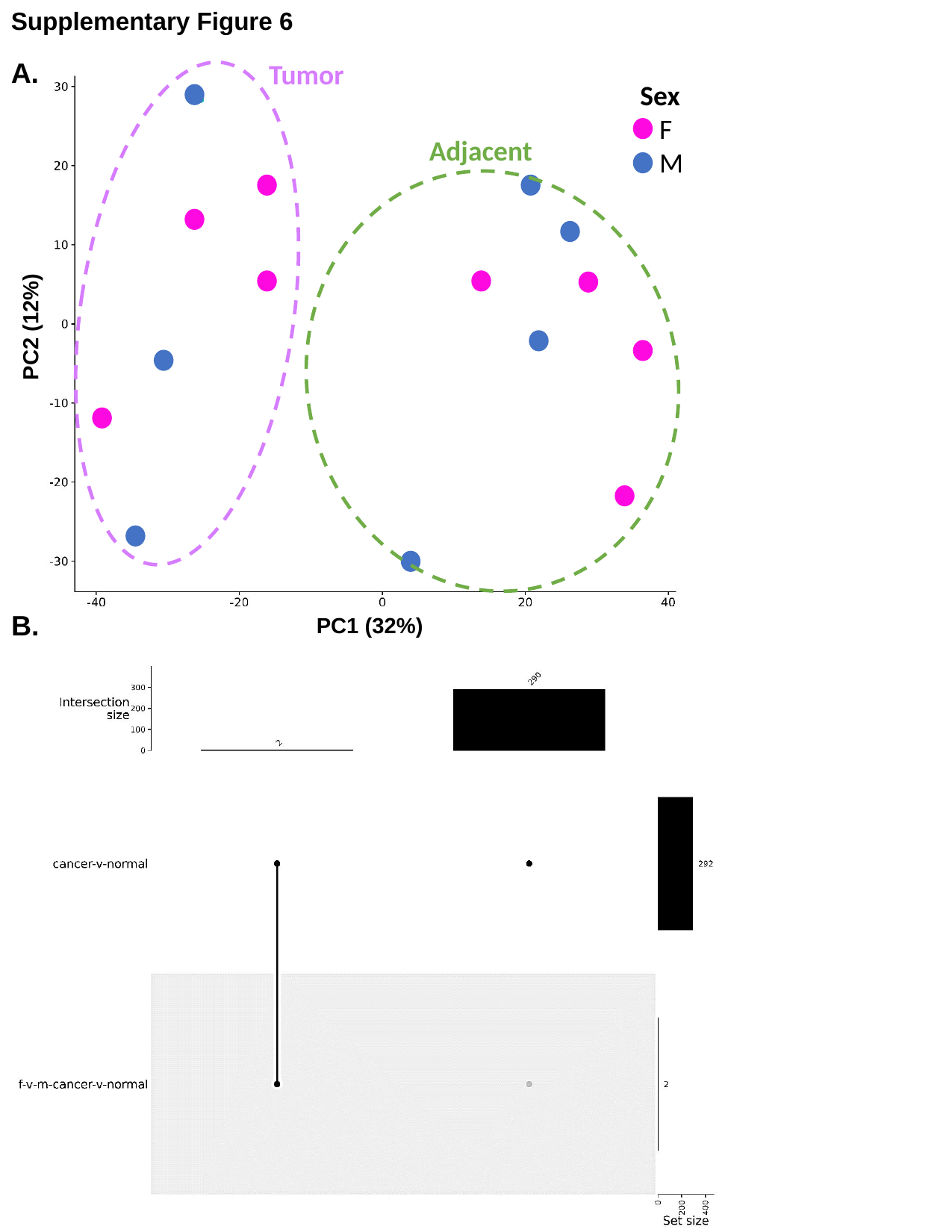

Supplementary Figure 6
A.
Tumor
 Sex
 F
 M
Adjacent
PC2 (12%)
B.
PC1 (32%)

## Slide 7
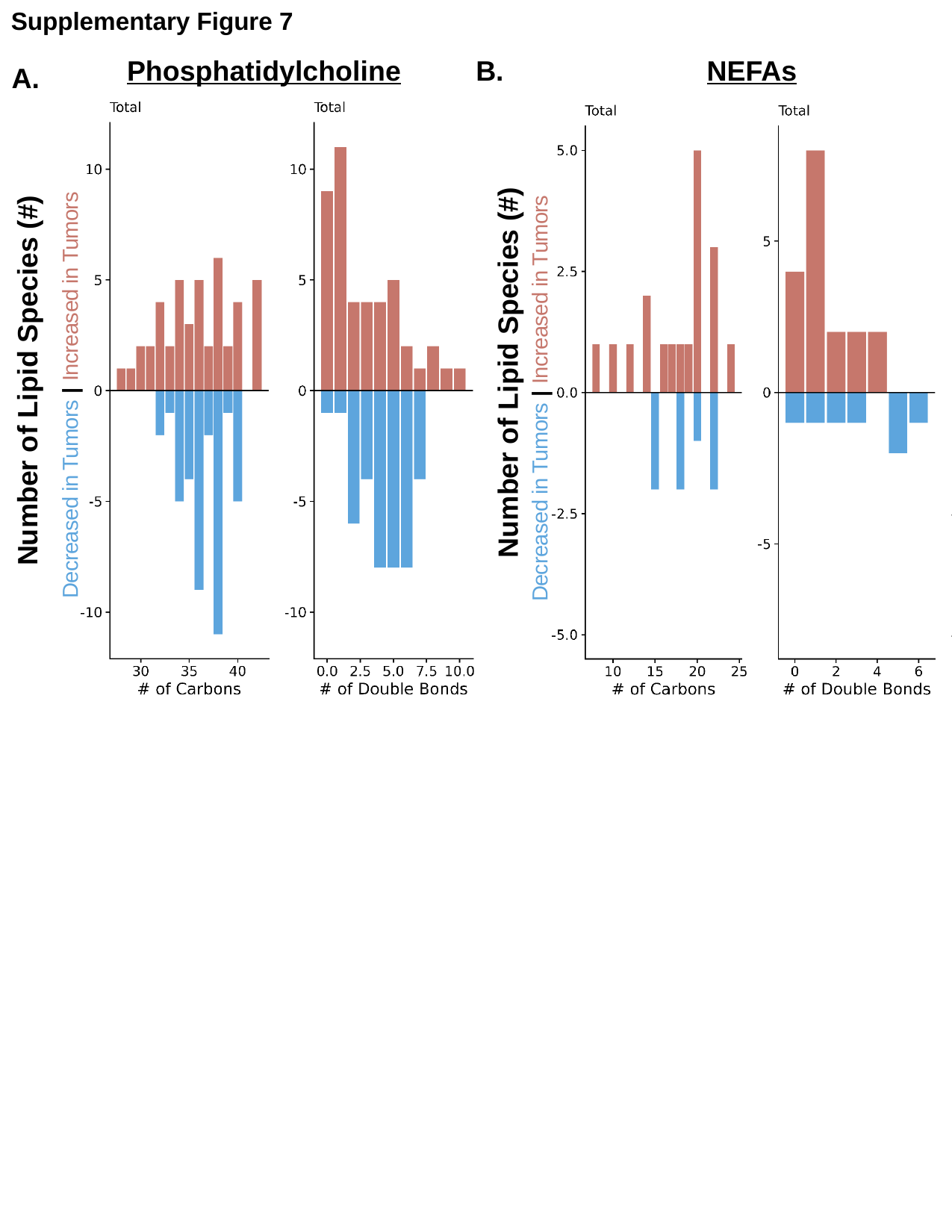

Supplementary Figure 7
NEFAs
Phosphatidylcholine
B.
A.
Number of Lipid Species (#)
Number of Lipid Species (#)
Decreased in Tumors | Increased in Tumors
Decreased in Tumors | Increased in Tumors

## Slide 8
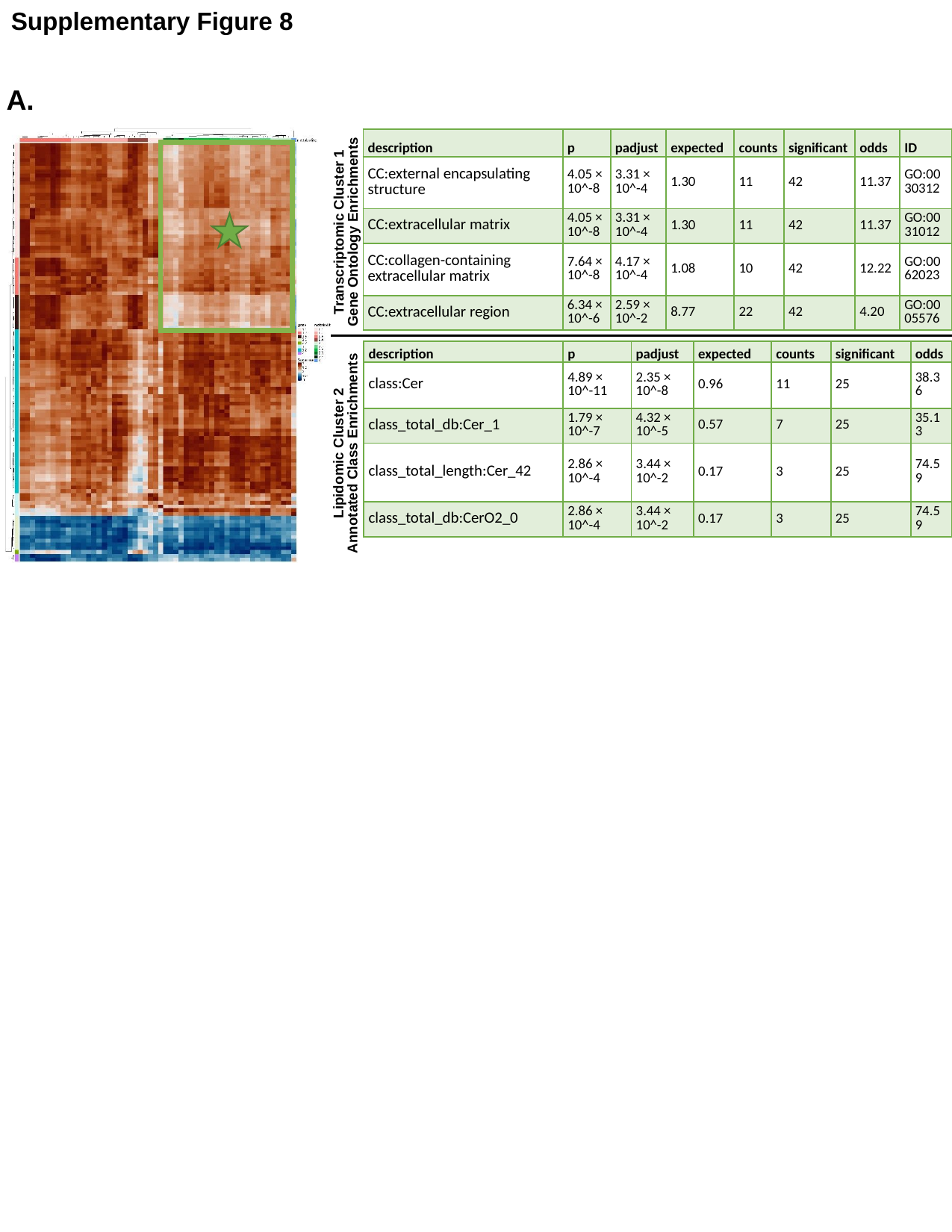

Supplementary Figure 8
A.
| description | p | padjust | expected | counts | significant | odds | ID |
| --- | --- | --- | --- | --- | --- | --- | --- |
| CC:external encapsulating structure | 4.05 × 10^-8 | 3.31 × 10^-4 | 1.30 | 11 | 42 | 11.37 | GO:0030312 |
| CC:extracellular matrix | 4.05 × 10^-8 | 3.31 × 10^-4 | 1.30 | 11 | 42 | 11.37 | GO:0031012 |
| CC:collagen-containing extracellular matrix | 7.64 × 10^-8 | 4.17 × 10^-4 | 1.08 | 10 | 42 | 12.22 | GO:0062023 |
| CC:extracellular region | 6.34 × 10^-6 | 2.59 × 10^-2 | 8.77 | 22 | 42 | 4.20 | GO:0005576 |
Transcriptomic Cluster 1
Gene Ontology Enrichments
| description | p | padjust | expected | counts | significant | odds |
| --- | --- | --- | --- | --- | --- | --- |
| class:Cer | 4.89 × 10^-11 | 2.35 × 10^-8 | 0.96 | 11 | 25 | 38.36 |
| class\_total\_db:Cer\_1 | 1.79 × 10^-7 | 4.32 × 10^-5 | 0.57 | 7 | 25 | 35.13 |
| class\_total\_length:Cer\_42 | 2.86 × 10^-4 | 3.44 × 10^-2 | 0.17 | 3 | 25 | 74.59 |
| class\_total\_db:CerO2\_0 | 2.86 × 10^-4 | 3.44 × 10^-2 | 0.17 | 3 | 25 | 74.59 |
Lipidomic Cluster 2
Annotated Class Enrichments

## Slide 9
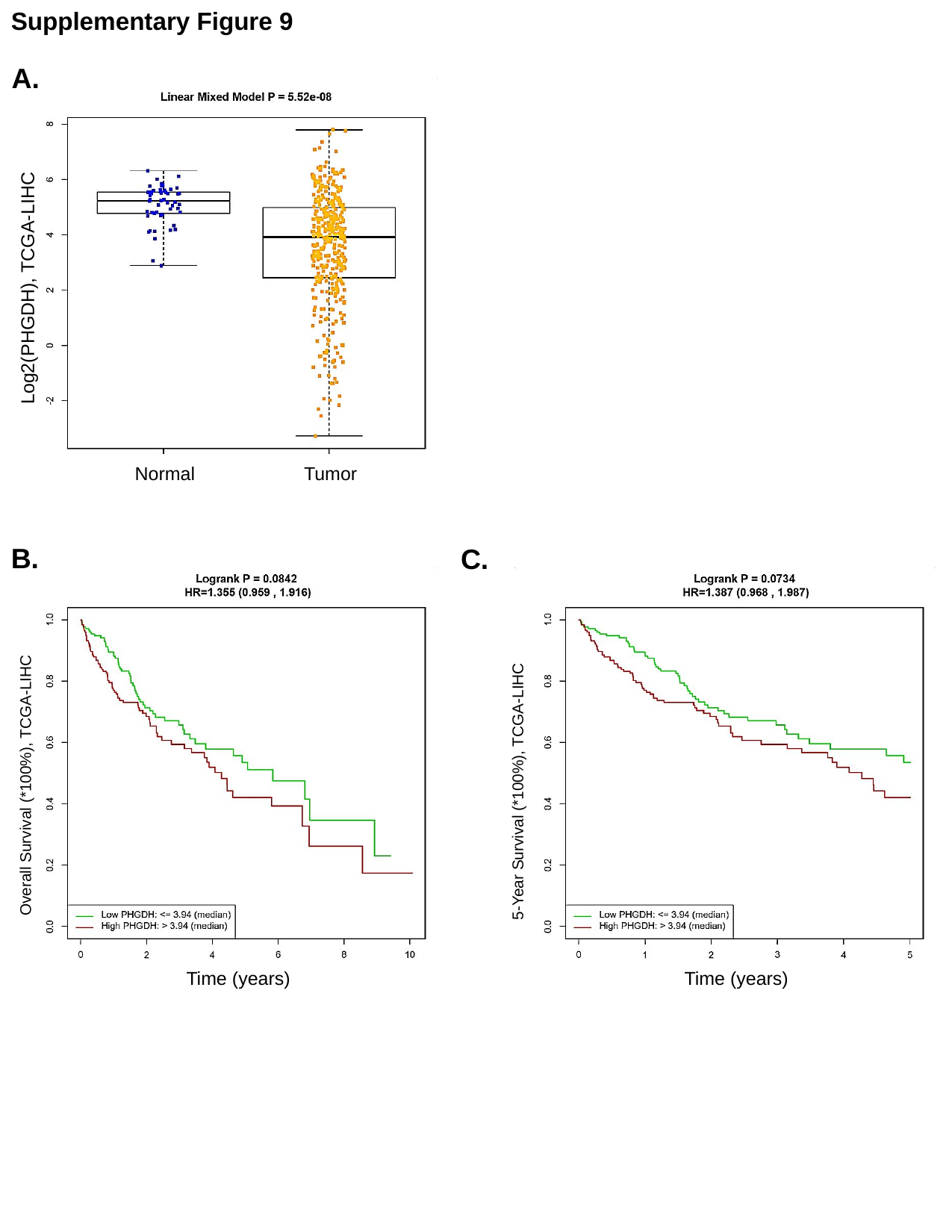

Supplementary Figure 9
A.
Log2(PHGDH), TCGA-LIHC
 Normal Tumor
B.
C.
 Overall Survival (*100%), TCGA-LIHC
 5-Year Survival (*100%), TCGA-LIHC
 Time (years)
 Time (years)

## Slide 10
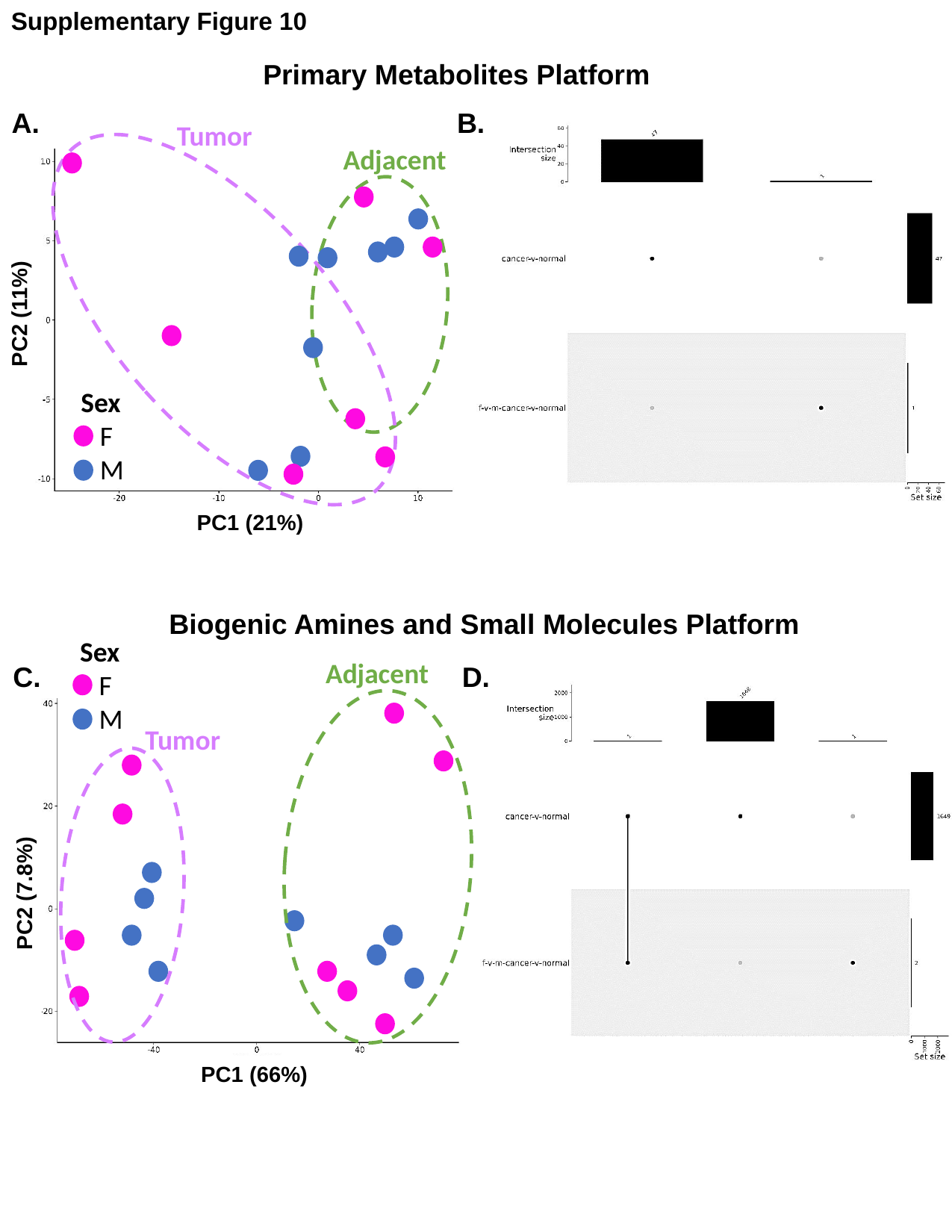

Supplementary Figure 10
Primary Metabolites Platform
A.
B.
Tumor
Adjacent
PC2 (11%)
 Sex
 F
 M
PC1 (21%)
Biogenic Amines and Small Molecules Platform
 Sex
 F
 M
Adjacent
C.
D.
Tumor
PC2 (7.8%)
PC1 (66%)

## Slide 11
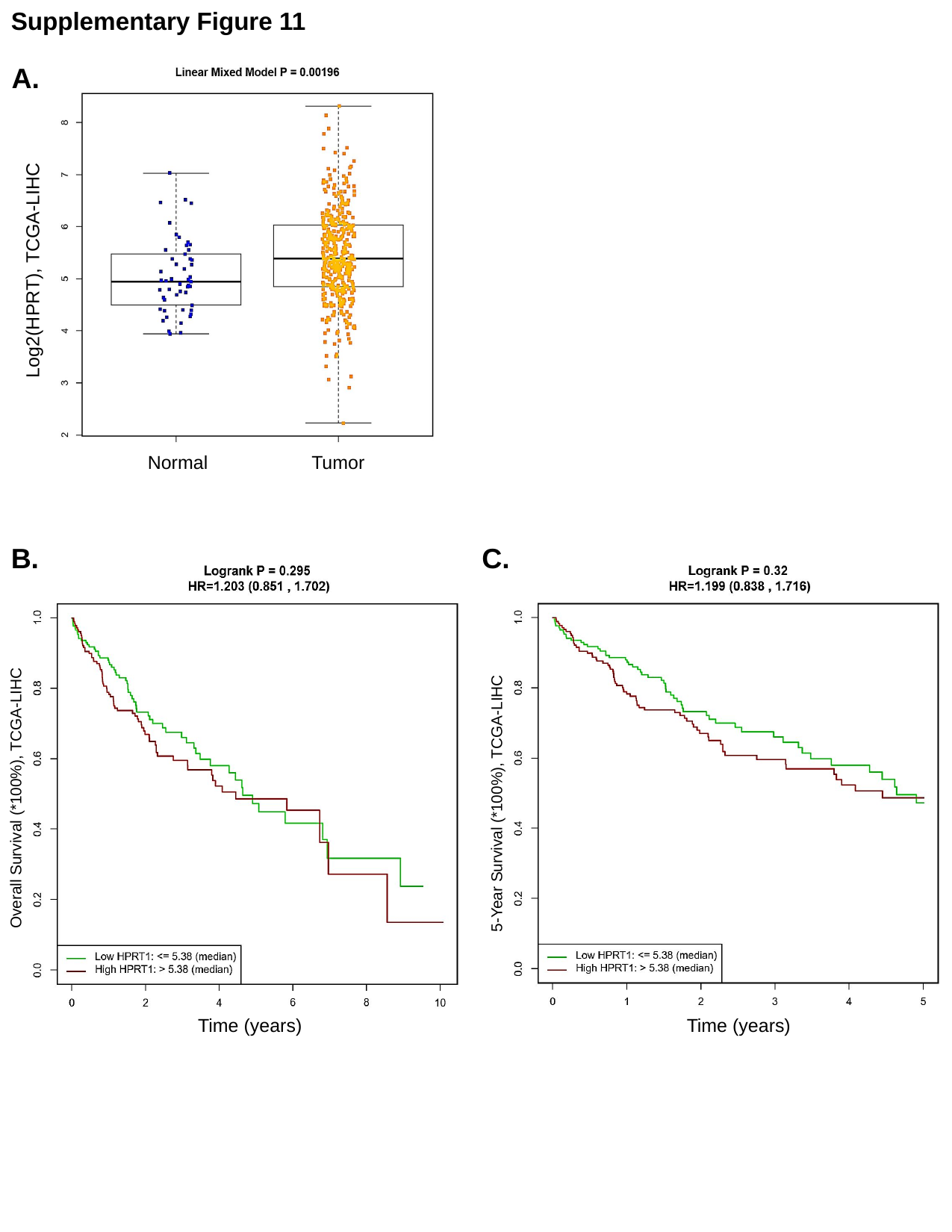

Supplementary Figure 11
A.
Log2(HPRT), TCGA-LIHC
 Normal Tumor
B.
C.
 Overall Survival (*100%), TCGA-LIHC
 5-Year Survival (*100%), TCGA-LIHC
 Time (years)
 Time (years)

## Slide 12
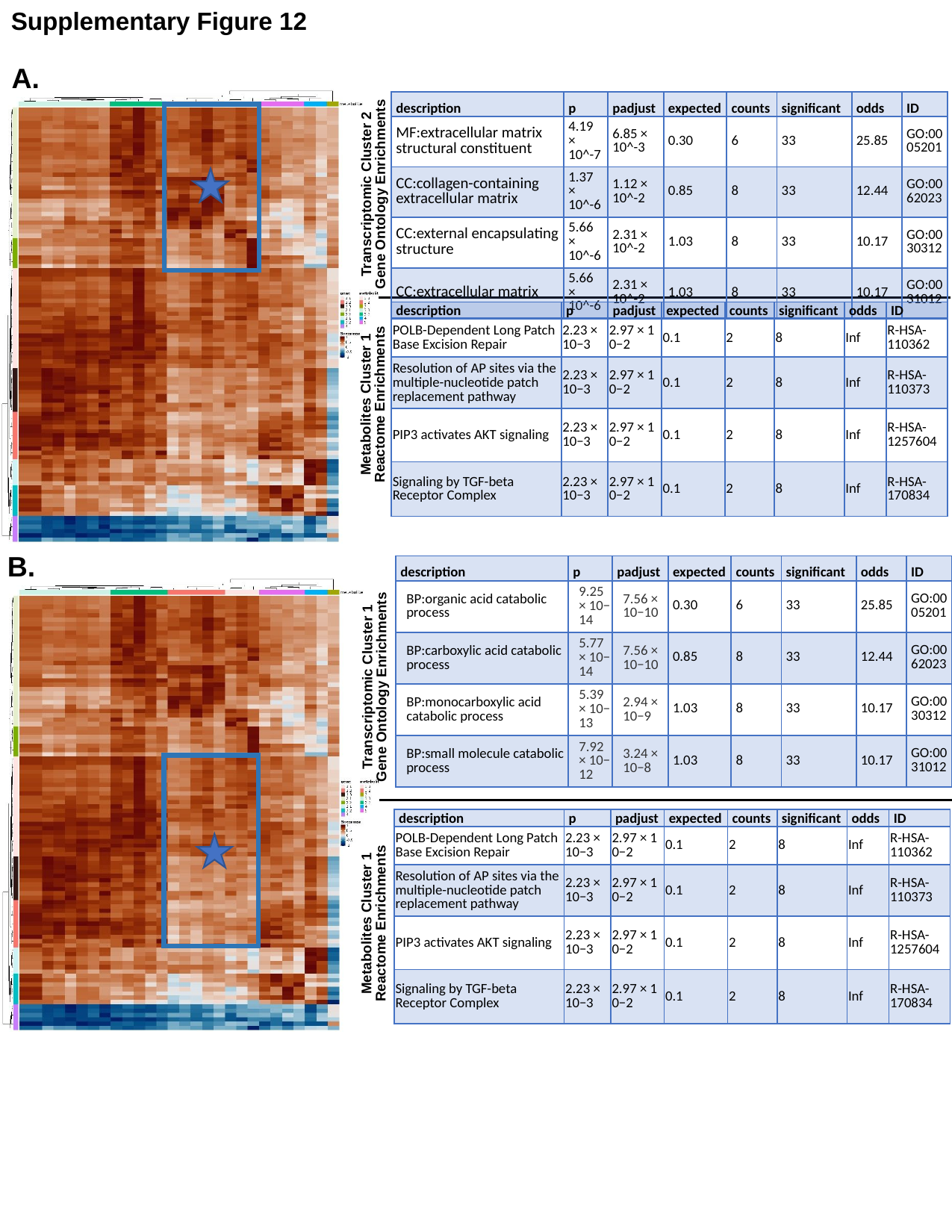

Supplementary Figure 12
A.
| description | p | padjust | expected | counts | significant | odds | ID |
| --- | --- | --- | --- | --- | --- | --- | --- |
| MF:extracellular matrix structural constituent | 4.19 × 10^-7 | 6.85 × 10^-3 | 0.30 | 6 | 33 | 25.85 | GO:0005201 |
| CC:collagen-containing extracellular matrix | 1.37 × 10^-6 | 1.12 × 10^-2 | 0.85 | 8 | 33 | 12.44 | GO:0062023 |
| CC:external encapsulating structure | 5.66 × 10^-6 | 2.31 × 10^-2 | 1.03 | 8 | 33 | 10.17 | GO:0030312 |
| CC:extracellular matrix | 5.66 × 10^-6 | 2.31 × 10^-2 | 1.03 | 8 | 33 | 10.17 | GO:0031012 |
Transcriptomic Cluster 2
Gene Ontology Enrichments
| description | p | padjust | expected | counts | significant | odds | ID |
| --- | --- | --- | --- | --- | --- | --- | --- |
| POLB-Dependent Long Patch Base Excision Repair | 2.23 × 10^−3^ | 2.97 × 10^−2^ | 0.1 | 2 | 8 | Inf | R-HSA-110362 |
| Resolution of AP sites via the multiple-nucleotide patch replacement pathway | 2.23 × 10^−3^ | 2.97 × 10^−2^ | 0.1 | 2 | 8 | Inf | R-HSA-110373 |
| PIP3 activates AKT signaling | 2.23 × 10^−3^ | 2.97 × 10^−2^ | 0.1 | 2 | 8 | Inf | R-HSA-1257604 |
| Signaling by TGF-beta Receptor Complex | 2.23 × 10^−3^ | 2.97 × 10^−2^ | 0.1 | 2 | 8 | Inf | R-HSA-170834 |
Metabolites Cluster 1
Reactome Enrichments
B.
| description | p | padjust | expected | counts | significant | odds | ID |
| --- | --- | --- | --- | --- | --- | --- | --- |
| BP:organic acid catabolic process | 9.25 × 10^−14^ | 7.56 × 10^−10^ | 0.30 | 6 | 33 | 25.85 | GO:0005201 |
| BP:carboxylic acid catabolic process | 5.77 × 10^−14^ | 7.56 × 10^−10^ | 0.85 | 8 | 33 | 12.44 | GO:0062023 |
| BP:monocarboxylic acid catabolic process | 5.39 × 10^−13^ | 2.94 × 10^−9^ | 1.03 | 8 | 33 | 10.17 | GO:0030312 |
| BP:small molecule catabolic process | 7.92 × 10^−12^ | 3.24 × 10^−8^ | 1.03 | 8 | 33 | 10.17 | GO:0031012 |
Transcriptomic Cluster 1
Gene Ontology Enrichments
| description | p | padjust | expected | counts | significant | odds | ID |
| --- | --- | --- | --- | --- | --- | --- | --- |
| POLB-Dependent Long Patch Base Excision Repair | 2.23 × 10^−3^ | 2.97 × 10^−2^ | 0.1 | 2 | 8 | Inf | R-HSA-110362 |
| Resolution of AP sites via the multiple-nucleotide patch replacement pathway | 2.23 × 10^−3^ | 2.97 × 10^−2^ | 0.1 | 2 | 8 | Inf | R-HSA-110373 |
| PIP3 activates AKT signaling | 2.23 × 10^−3^ | 2.97 × 10^−2^ | 0.1 | 2 | 8 | Inf | R-HSA-1257604 |
| Signaling by TGF-beta Receptor Complex | 2.23 × 10^−3^ | 2.97 × 10^−2^ | 0.1 | 2 | 8 | Inf | R-HSA-170834 |
Metabolites Cluster 1
Reactome Enrichments

## Slide 13
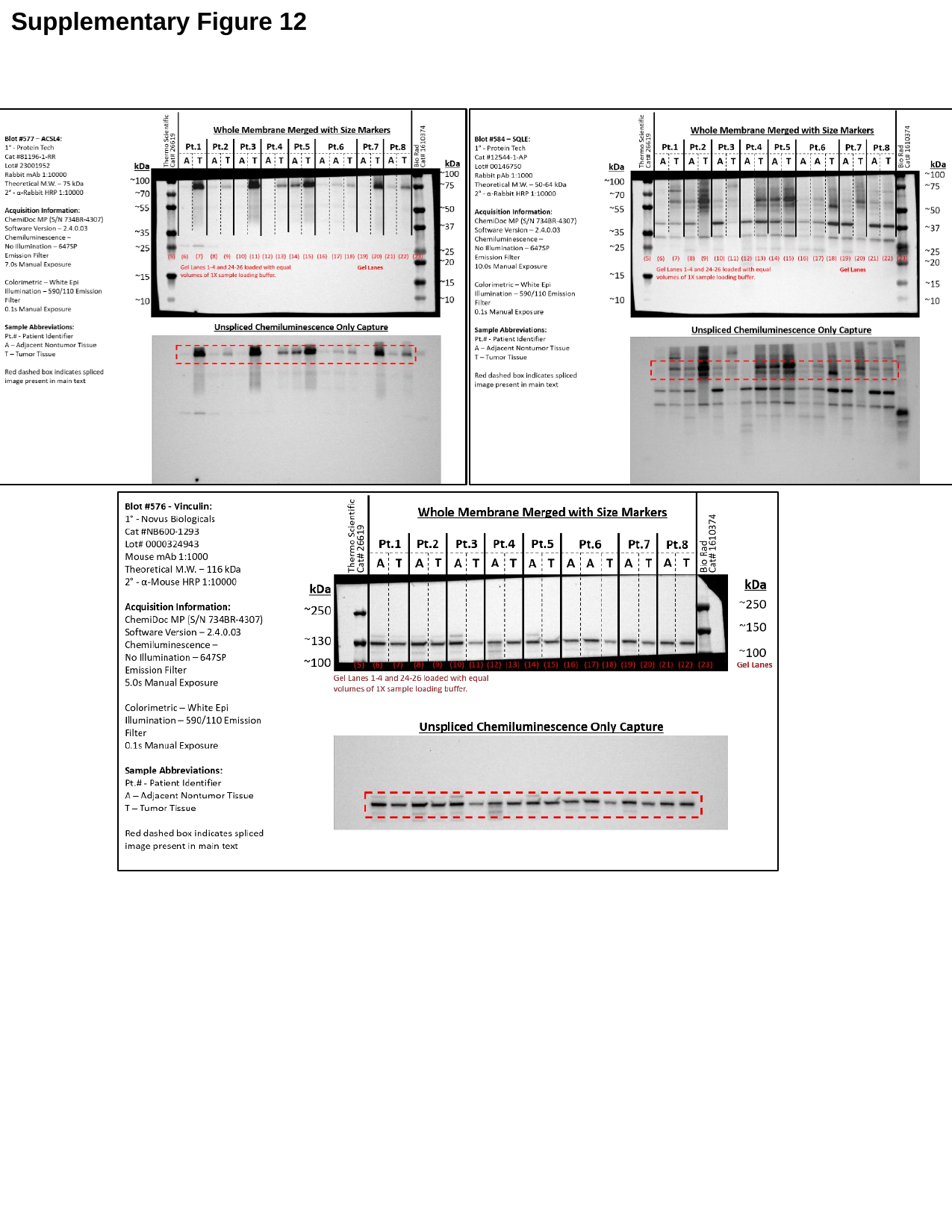

Supplementary Figure 12
